# Supplementary material for: Convergence of two global regulators to coordinate expression of essential virulence determinants of Mycobacterium tuberculosis
Source: eLife. 2022 Nov 9;11:e80965. doi: 10.7554/eLife.80965 (PMC9645806; doi:10.7554/eLife.80965)
Supplement: Supplementary file 1. — (a) Oligonucleotide primers used for amplification and cloning in this study. (b) Plasmids used in this study. (c) Sequence of oligonucleotide primers used in aRT-qPCR and bChIP-qPCR experiments reported in this study. [file elife-80965-supp1.docx]

Convergence of two global regulators to coordinate expression of essential virulence determinants of *Mycobacterium tuberculosis*

Hina Khan^1,2^, Partha Paul^1^, Ritesh Rajesh Sevalkar^1,3^, Sangita Kachhap^1,4^, Balvinder Singh^1^ and Dibyendu Sarkar^1,^ *

^1^CSIR-Institute of Microbial Technology, Sector 39 A, Chandigarh 160036, India

Running title: Regulation of mycobacterial virulence determinants

Key Words: CRP; CRP-PhoP interaction; *Mycobacterium tuberculosis* PhoP;

transcription factors, virulence determinants

^2^Present address: Department of Biosciences and Bioengineering,

Indian Institute of Technology Roorkee, Uttarakhand, India

^3^Present address: Department of Microbiology, University of Alabama,

Birmingham, Alabama, United States

^4^Present address: Jerzy Haber Institute of Catalysis and Surface Chemistry

Polish Academy of Sciences, Niezapominajek 8,

PL- 30239 Krakow, Poland

*Address correspondence to: Dibyendu Sarkar, CSIR-Institute of Microbial Technology,

Tel.: 091-172-6665291; Fax: 091-172-2690585; E-mail: [dibyendu@imtech.res.in](mailto:dibyendu@imtech.res.in)

Supplementary file 1a

Oligonucleotide primers used for amplification and cloning in this study

| **Primers^a^** | **Sequences (5’-3’)** | **Reference** |
| --- | --- | --- |
| FPwhiB1^up^ | AATAATAAGCTTAGATGTGATGGG | This study |
| RPwhiB1^up^ | AATAATGGTACCTACCGGGAAGAA | This study |
| FPwhiB1^upmut^ | GTCAAACAAGGATCACAAAACGAGATCGCCA | This study |
| RPwhiB1^upmut^ | TTGTGATCCTTGTTTGACTGTACTACACTA | This study |
| FPwhiB1^up1^ | AATAATAAGCTTAGATGTGATGGG | This study |
| RPwhiB1^up1^ | AATAATGGTACCAGCGCGTGGGCT | This study |
| FPwhiB1^up2^ | AATAATGGATCCTACGTAACACTA | This study |
| RPwhiB1^up2^ | AATAATGGTACCAGCGCGTGGGCT | This study |
| FPwhiB1^up3^ | AATAATTCCGAAGAAACGCCT | This study |
| FPphoPstart | AATAATGGATCCATGCGGAAAGGGGT | This study |
| RPphoPFLAG | AATAAGCTTTCACTTGTCGTCATCGTCTTTGTAGTCTCGAGGCTCCCGCA | This study |
| FPmCMR | AATAATTGTTCGACTGACG | This study |
| RPmCMR | AATAATCGGGTTTGTGTTGT | This study |
| FPmCRP | AATAATAAGCTTGTGGACGAGATC | This study |
| RPmCRP | AATAATATCGATTTACCTCGCTCG | This study |
| FPPhoP^N^ | CCTGGATCCATGCGGAAAGGGGTT | ([Pathak et al., 2010](#_ENREF_7)) |
| RPPhoP^N^ | AATAATCTCGAGGCGTCGCAGGATGA | This study |
| FPPhoP^C^ | AATAATCGGATCCAAGGGCAACAAGGAACCA | This study |
| RPPhoP^C^ | GGTCTCGAGTCGAGGCTCCCGCAG | ([Pathak et al., 2010](#_ENREF_7)) |
| FPCRP | AATAATCATATGGTGGACGAGATC | This study |
| FPCRP^N^ | ATATATGGATCCCCCGTCGACTTCCCCC | This study |
| RPCRP^N^ | ATATATAAGCTTTCAACGATCGGCGAT | This study |
| FPCRP^C^ | ATATATCATATGGTGCCCGGTCGGGT | This study |
| RPCRP | AATAATCTCGAGTTACCTCGCTCG | This study |
| FPCRPG79A | CATGTTCGCGGAGTTGTCGATCT | This study |
| RPCRPG79A | CGACAACTCCGCGAACATGTCCGA | This study |
| FPCRPT90A | GTCCGCGCGCGTCCAGC | This study |
| RPCRPT90A | GCTGGACGCGCGCGGACCCGGGT | This study |
| FPPhoP62-64A | GCAGCAGCACCGGACGCGGTG | This study |
| RPPhoP62-64A | TGCTGCTGCCCGGGCCCGATC | This study |
| FPPhoP76-78A | GCAGCAGCAGGCTTTGGGGTG | This study |
| RPPhoP76-78A | TGCTGCTGCGGGCATCATCAC | This study |
| FPPhoP105-107A | GCAGCAGCAATCGCGGGTCTG | This study |
| RPPhoP105-107A | TGCTGCTGCTAGCGAGTCACG | This study |
| FPPhoP110-112A | GCAGCAGCACTGGGTGGTGAC | This study |
| RPPhoP110-112A | TGCTGCTGCCGCGATCTTGTC | This study |
| FPPhoP118-120A | GCAGCAGCAAAGCCCTTCAGT | This study |
| RPPhoP118-120A | TGCTGCTGCGTCGTCACCACC | This study |

^a^FP, forward primer; RP, reverse primer

Supplementary file 1b

Plasmids used in this study

| **Plasmids** |  |  |
| --- | --- | --- |
| pET-*phoP*^a^ | His_6_ tagged-PhoP residues 1-247 cloned in pET15b | ([Gupta et al., 2009](#_ENREF_6)) |
| pGEX-*phoP* | PhoP residues 1-247 cloned in pGEX-4T-1 | ([Gupta et al., 2009](#_ENREF_6)) |
| pGEX-*phoP*LAla5 | G142-P146 residues mutated to A in *phoP* of pGEX-*phoP* | This study |
| pGEX-*phoP^N^* | PhoP residues 1-141 cloned in pGEX-4T-1 | This study |
| pGEX-*phoP^C^* | PhoP residues 141-247 cloned in pGEX-4T-1 | This study |
| pGEX-*phoP*(62-64)Ala | E62-R64 residues mutated to A in *phoP* of pGEX-*phoP* | This study |
| pGEX-*phoP*(76-78)Ala | G76-D78 residues mutated to A in *phoP* of pGEX-*phoP* | This study |
| pGEX-*phoP*(105-107)Ala | Q105-K107 residues mutated to A in *phoP* of pGEX-*phoP* | This study |
| pGEX-*phoP*(110-112)Ala | G110-T112 residues mutated to A in *phoP* of pGEX-*phoP* | This study |
| pGEX-*phoP*(118-120)Ala | Y118-T120 residues mutated to A in *phoP* of pGEX-*phoP* | This study |
| pME1mL1-*phoP*^b^ | PhoP residues 1-247 cloned in pME1mL1 | ([Goyal et al., 2011](#_ENREF_5)) |
| pSM128^c^ | Integrative promoter probe vector for mycobacteria | ([Dussurget et al., 1999](#_ENREF_4)) |
| pSM-*whiB1*^up^ | whiB1up-*lacZ* fusion in pSM128 | This study |
| pSM-*whiB1*^upmut^ | pSM-*whiB1*up carrying changes in the PhoP binding site | This study |
| pUAB400^d^ | Integrative mycobacteria - *E. coli* shuttle plasmid, Kan^r^ | ([Singh et al., 2006](#_ENREF_8)) |
| pUAB400-*phoP* | PhoP residues 1-247 cloned in pUAB400 | ([Singh et al., 2014](#_ENREF_9)) |
| pUAB300^b^ | Episomal mycobacteria - *E. coli* shuttle plasmid, Hyg^r^ | ([Singh et al., 2006](#_ENREF_8)) |
| pUAB300-*crp* | CRP residues 1-224 cloned in pUAB300 | This study |
| pUAB300-*cmr* | CMR residues 1-244 cloned in pUAB300 | This study |
| pET-28c^d^ | *E. coli* cloning vector, Kan^r^ | Novagen |
| pET-*crp*^d^ | His_6_ tagged-CRP residues 1-224 cloned in pET-28c | This study |
| pET-*crp^N^* | His_6_ tagged -CRP residues 28-116 cloned in pET-28c | This study |
| pET-*crp^C^* | His_6_ tagged-CRP residues 146-224 cloned in pET-28c | This study |
| p19Kpro^b^ | Mycobacteria expression vector | ([De Smet et al., 1999](#_ENREF_3)) |
| p19Kpro-*phoP* | His_6_-tagged PhoP residues 1-247 cloned in p19Kpro | ([Anil Kumar et al., 2016](#_ENREF_1)) |
| p19Kpro-*phoP*^FLAG^ | FLAG-tagged PhoP residues 1-247 cloned in p19Kpro | This study |

^a^ ampicillin resistance; ^b^ hygromycin resistance

^c^ streptomycin resistance; ^d^ kanamycin resistance

Supplementary file 1c

Sequence of oligonucleotide primers used in ^a^RT-qPCR and ^b^ChIP-qPCR experiments reported in this study

| Primers^a^ | Sequences (5’-3’) | Reference |
| --- | --- | --- |
| FPaprART | TTGACCATGACAGCGAGTGT | ([Bansal et al., 2017](#_ENREF_2)) |
| RPaprART | TTGGACAGAAATGCAGGATG | ([Bansal et al., 2017](#_ENREF_2)) |
| FPcrpRT | ATCATCATCTCGGGGAAGGT | This study |
| RPcrpRT | CAGCTGTTCGGAGATTTCG | This study |
| FPcmrRT | ATTGGCCGAAACGTTACAAG | This study |
| RPcmrRT | ACCATCGGCATCTCCAGTAG | This study |
| FPicl1RT | GCTTCTACCGCACCAAGAAC | This study |
| RPicl1RT | TCGAGGTGCTTTTTCCAGTT | This study |
| FPphoPRT | GCCTCAAGTTCCAGGGCTTT | This study |
| RPphoPRT | CCGGGCCCGATCCA | This study |
| FPumaART | CGTTATGCGGCATTCTTTG | This study |
| RPumaART | TGCGCAAATTTGAAGATGTC | This study |
| FPwhiB1RT | CACAAGGCGGTCTGTCGT | This study |
| RPwhiB1RT | GAGTCCTGGCCGGTATTCAG | This study |
| FPrpoBRT | GGAGGCGATCACACCGCAGACGTT | This study |
| RPrpoBRT | CCTCCAGCCCGGCACGCTCACGT | This study |
| FP16SrDNART | CTGAGATACGGCCCAGACTC | This study |
| RP16SrDNART | CGTCGATGGTGAAAGAGGTT | This study |
| Primers^b^ |  |  |
| FPespA^up^ | CGTGATCTTGATACGGCTCG | ([Anil Kumar et al., 2016](#_ENREF_1)) |
| RPespA^up^ | GTTGTTGGTACCCTCGGCAAGATCGGC | ([Anil Kumar et al., 2016](#_ENREF_1)) |
| FPgapdh^up^ | GAGTAGGCATCAACGGGTTTG | This study |
| RPgapdh^up^ | GTGCTGTTGTCGGTGATGTC | This study |
| FPicl1^up^ | AATAATAAGCTTACCGGATCCGCA | This study |
| RPicl1^up^ | AATAATGGTACCGTTCGTGTCC | This study |
| FP16SrDNA^up^ | CTGAGATACGGCCCAGACTC | ([Singh et al., 2014](#_ENREF_9)) |
| RP16SrDNA^up^ | CGTCGATGGTGAAAGAGGTT | ([Singh et al., 2014](#_ENREF_9)) |
| FPsucC^up^ | GGCTGTGATTGTGAGTTGGA | This study |
| RPsucC^up^ | GCGAATAACTCCTTGGCTTG | This study |
| FPumaA^up^ | TGTTGCTGCGTATGGTTGAG | This study |
| RPumaA^up^ | AATCGATTGCGACTCTTCGT | This study |
| FPwhiB1^up1^ | AATAATAAGCTTAGATGTGATGGG | This study |
| RPwhiB1^up1^ | AATAATGGTACCAGCGCGTGGGCT | This study |

^a^FP, forward primer; RP, reverse primer

**References**

Anil Kumar, V., Goyal, R., Bansal, R., Singh, N., Sevalkar, R.R., Kumar, A., and Sarkar, D. (2016). EspR-dependent ESAT-6 Protein Secretion of Mycobacterium tuberculosis Requires the Presence of Virulence Regulator PhoP. J Biol Chem *291*, 19018-19030.

Bansal, R., Anil Kumar, V., Sevalkar, R.R., Singh, P.R., and Sarkar, D. (2017). Mycobacterium tuberculosis virulence-regulator PhoP interacts with alternative sigma factor SigE during acid-stress response. Mol Microbiol *104*, 400-411.

De Smet, K.A., Kempsell, K.E., Gallagher, A., Duncan, K., and Young, D.B. (1999). Alteration of a single amino acid residue reverses fosfomycin resistance of recombinant MurA from Mycobacterium tuberculosis. Microbiology *145 ( Pt 11)*, 3177-3184.

Dussurget, O., Timm, J., Gomez, M., Gold, B., Yu, S., Sabol, S.Z., Holmes, R.K., Jacobs, W.R., Jr., and Smith, I. (1999). Transcriptional control of the iron-responsive fxbA gene by the mycobacterial regulator IdeR. J Bacteriol *181*, 3402-3408.

Goyal, R., Das, A.K., Singh, R., Singh, P.K., Korpole, S., and Sarkar, D. (2011). Phosphorylation of PhoP protein plays direct regulatory role in lipid biosynthesis of Mycobacterium tuberculosis. J Biol Chem *286*, 45197-45208.

Gupta, S., Pathak, A., Sinha, A., and Sarkar, D. (2009). Mycobacterium tuberculosis PhoP recognizes two adjacent direct-repeat sequences to form head-to-head dimers. J Bacteriol *191*, 7466-7476.

Pathak, A., Goyal, R., Sinha, A., and Sarkar, D. (2010). Domain structure of virulence-associated response regulator PhoP of Mycobacterium tuberculosis: role of the linker region in regulator-promoter interaction(s). J Biol Chem *285*, 34309-34318.

Singh, A., Mai, D., Kumar, A., and Steyn, A.J. (2006). Dissecting virulence pathways of Mycobacterium tuberculosis through protein-protein association. Proc Natl Acad Sci U S A *103*, 11346-11351.

Singh, R., Anil Kumar, V., Das, A.K., Bansal, R., and Sarkar, D. (2014). A transcriptional co-repressor regulatory circuit controlling the heat-shock response of Mycobacterium tuberculosis. Mol Microbiol *94*, 450-465.
