# Supplementary figures and images for: Convergence of two global regulators to coordinate expression of essential virulence determinants of Mycobacterium tuberculosis
Source: eLife. 2022 Nov 9;11:e80965. doi: 10.7554/eLife.80965 (PMC9645806; doi:10.7554/eLife.80965)

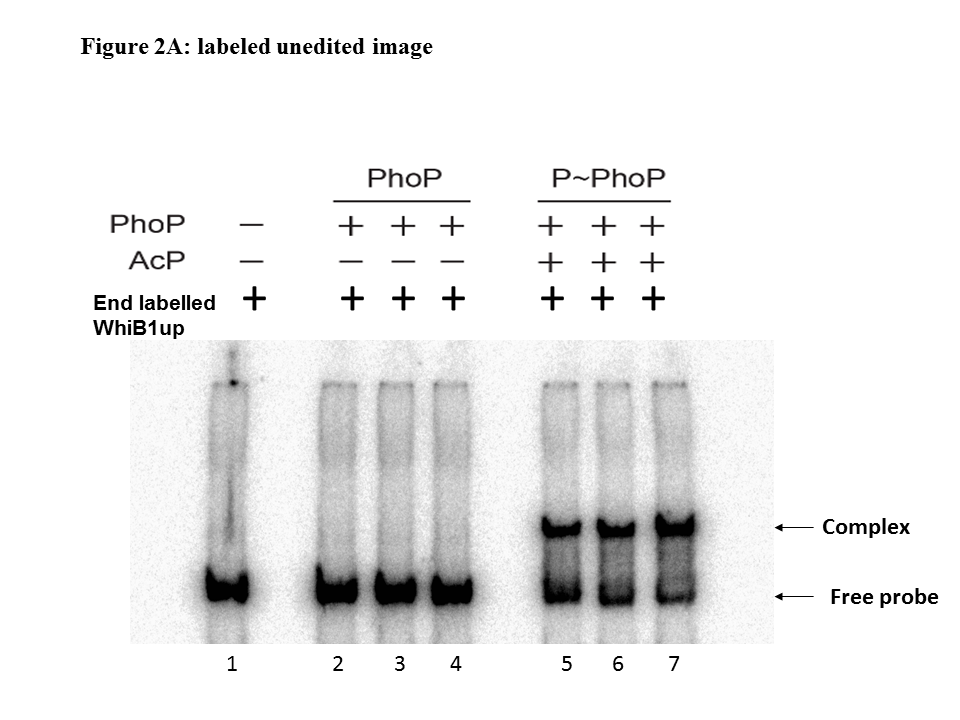

Supplement: Source data 1. [file elife-80965-data1.zip › Source Data File October 2022/Figure 2A-labeled unedited image.tif]

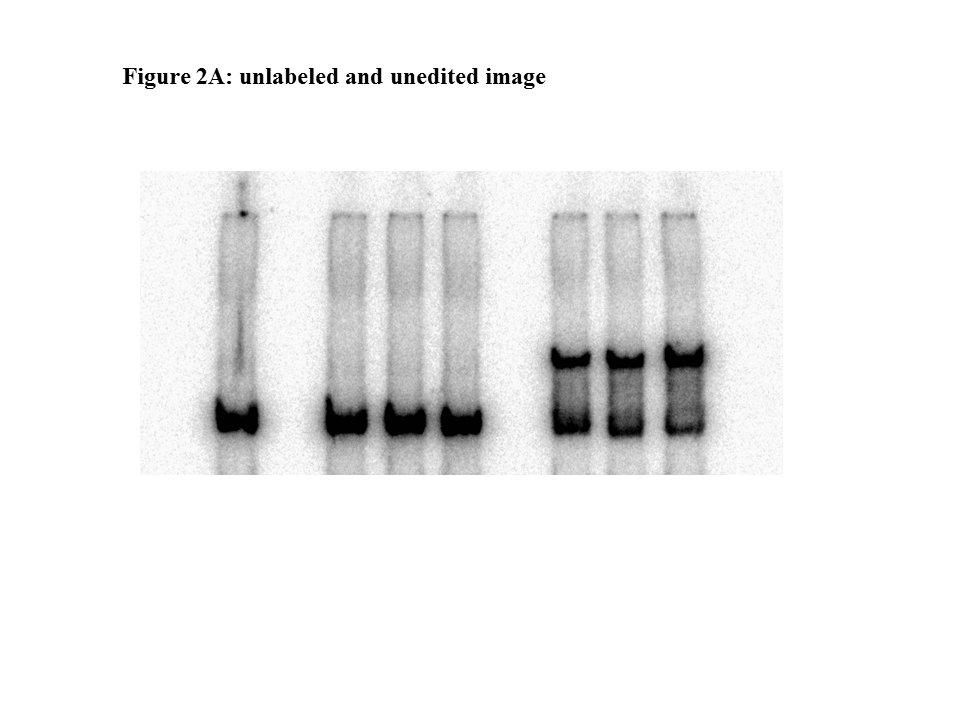

Supplement: Source data 1. [file elife-80965-data1.zip › Source Data File October 2022/Figure 2A-unlabeled and unedited image.tif]

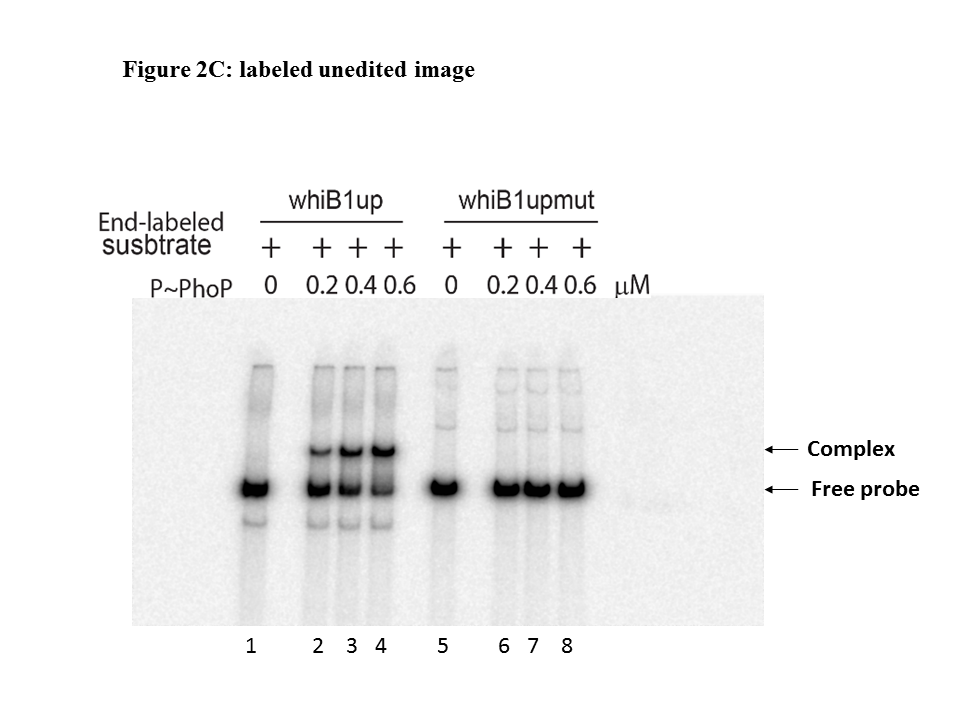

Supplement: Source data 1. [file elife-80965-data1.zip › Source Data File October 2022/Figure 2C-labeled unedited image.tif]

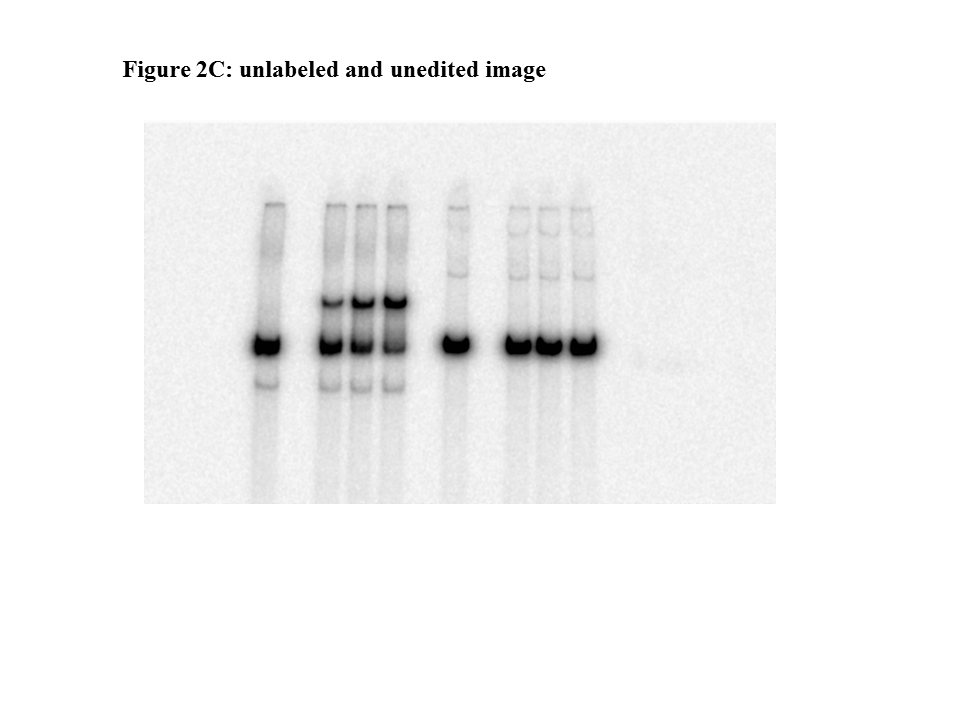

Supplement: Source data 1. [file elife-80965-data1.zip › Source Data File October 2022/Figure 2C-unlabeled and unedited image.tif]

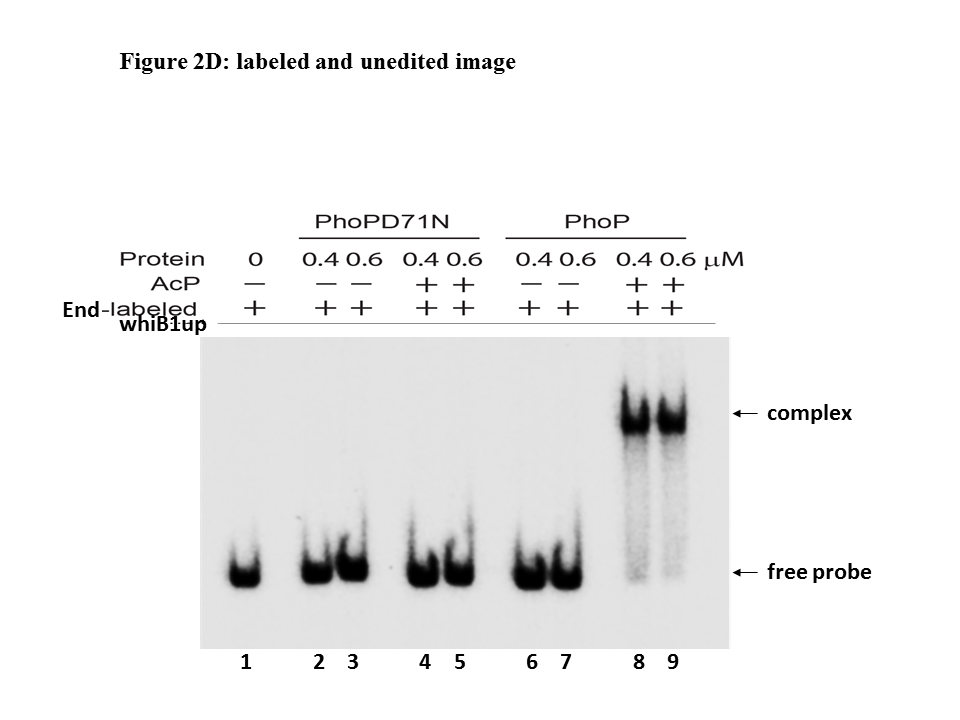

Supplement: Source data 1. [file elife-80965-data1.zip › Source Data File October 2022/Figure 2D- labeled unedited image.tif]

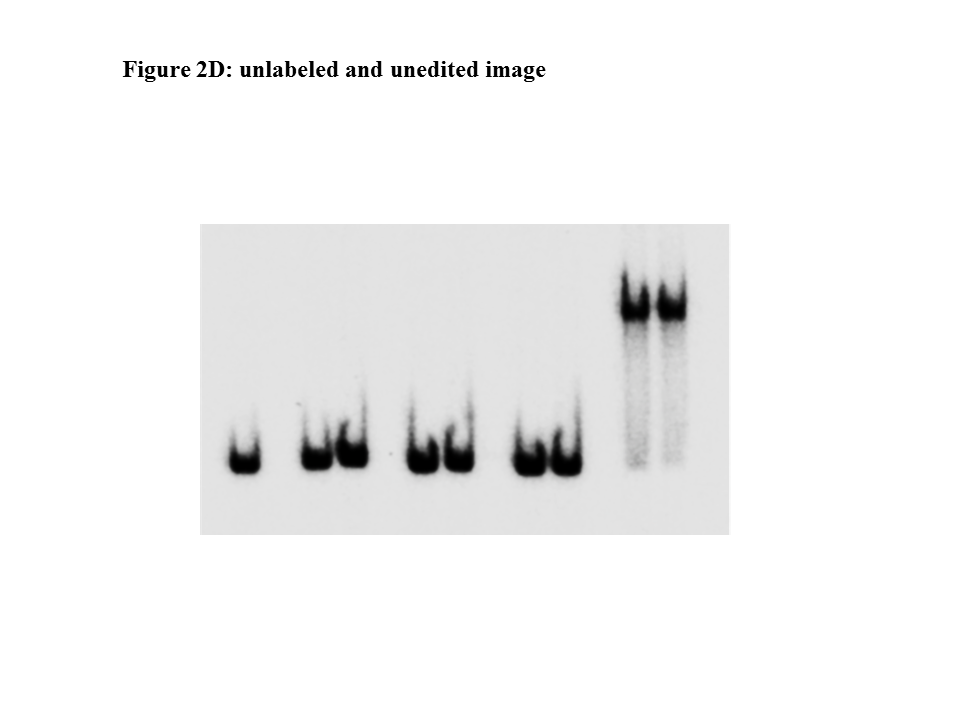

Supplement: Source data 1. [file elife-80965-data1.zip › Source Data File October 2022/Figure 2D- unlabeled and unedited image.tif]

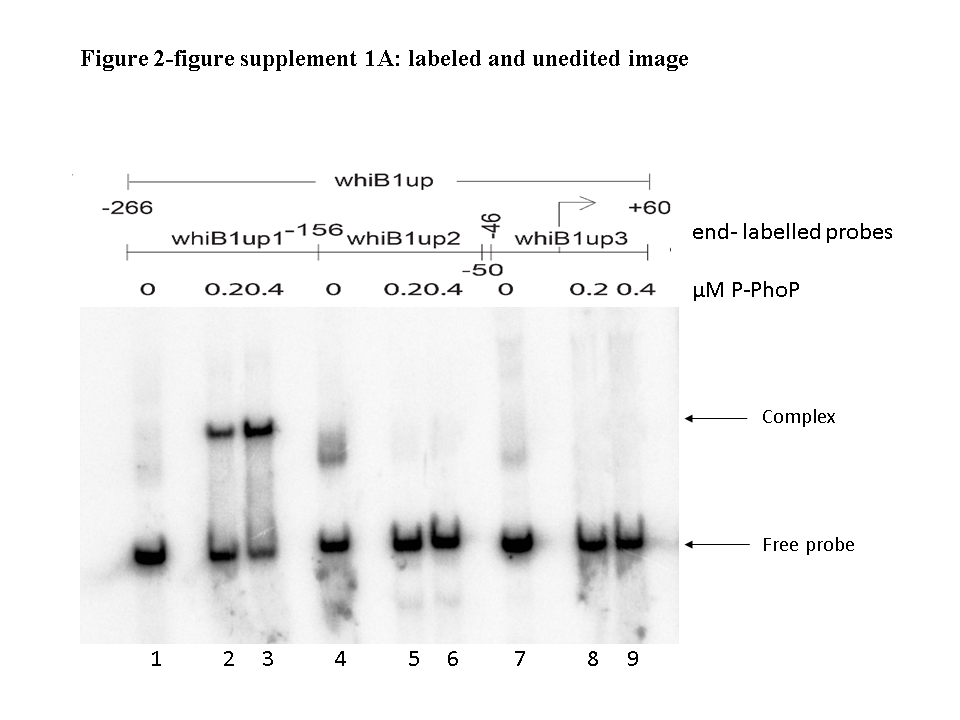

Supplement: Source data 1. [file elife-80965-data1.zip › Source Data File October 2022/Figure 2-figure supplement 1A-labeled and unedited image.tif]

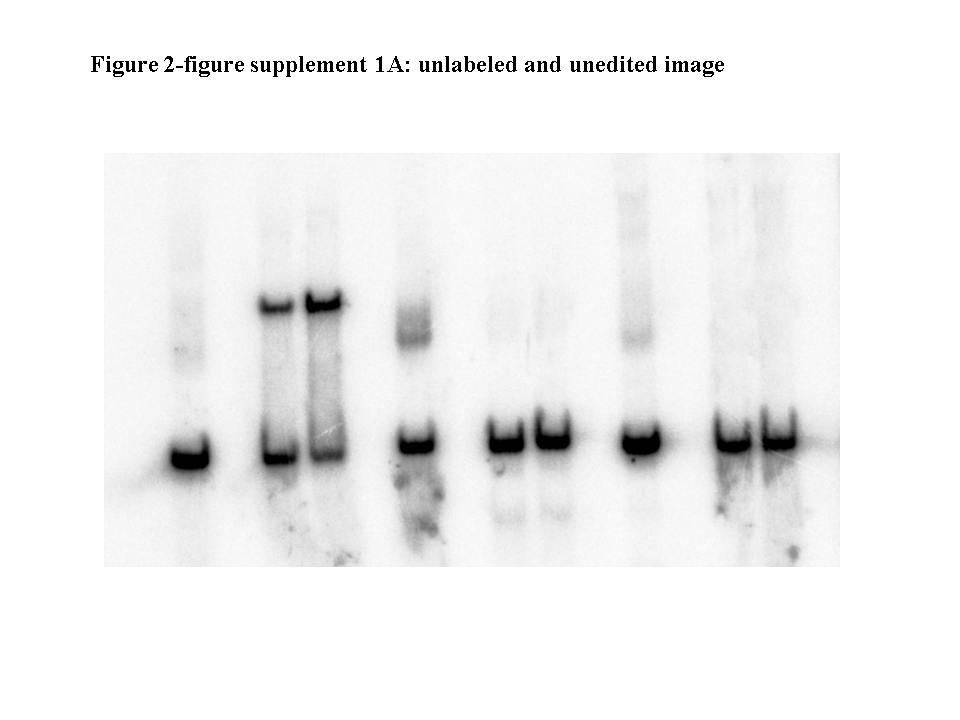

Supplement: Source data 1. [file elife-80965-data1.zip › Source Data File October 2022/Figure 2-figure supplement 1A-unlabeled and unedited image.tif]

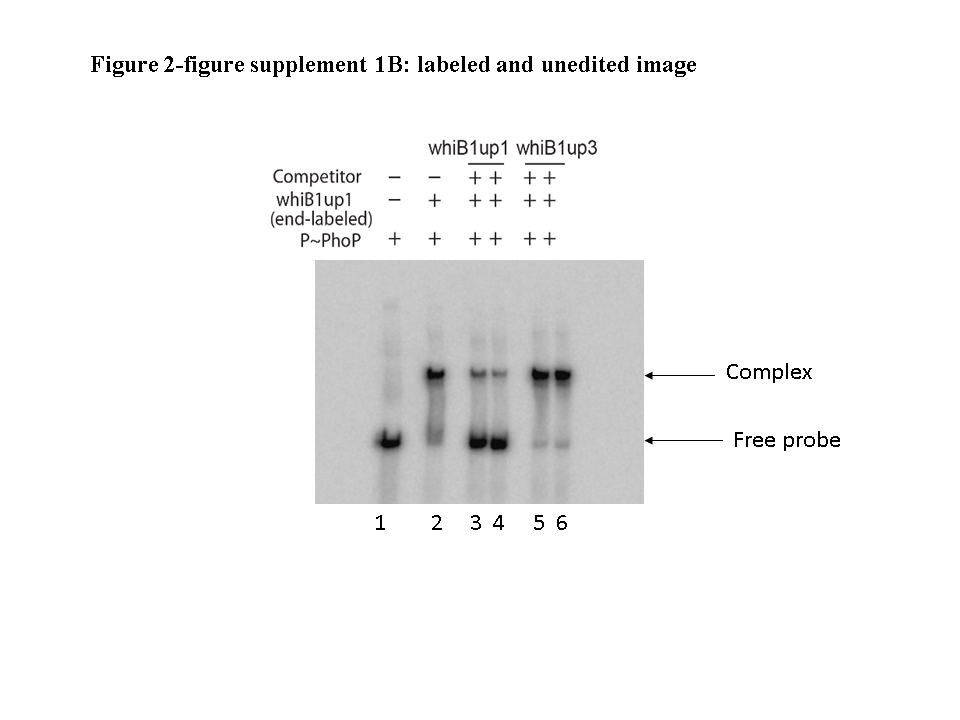

Supplement: Source data 1. [file elife-80965-data1.zip › Source Data File October 2022/Figure 2-figure supplement 1B-labeled and unedited image.tif]

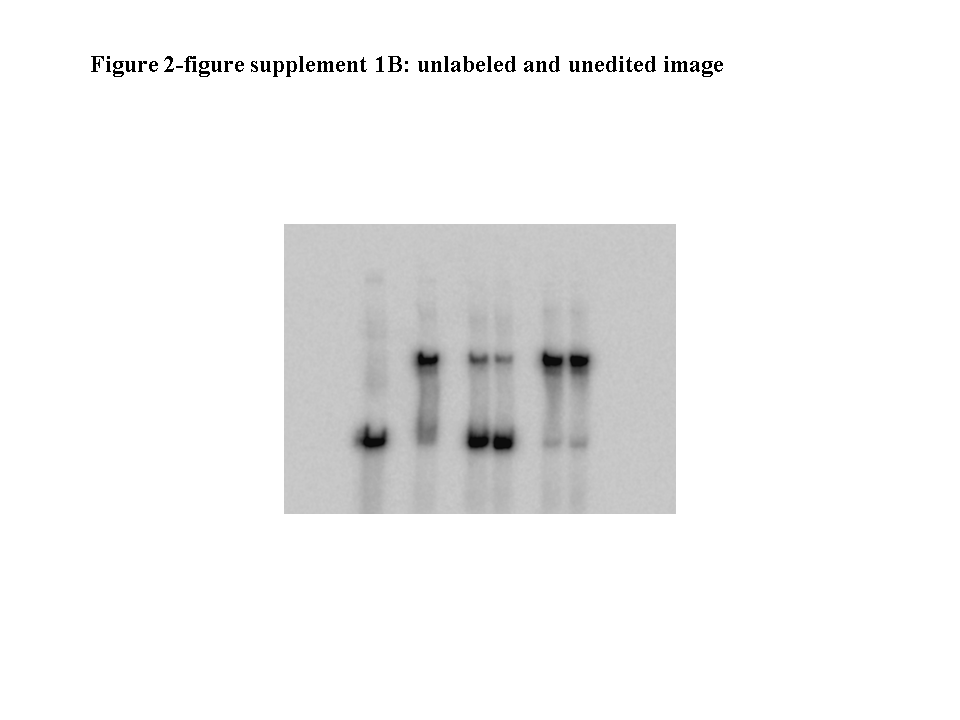

Supplement: Source data 1. [file elife-80965-data1.zip › Source Data File October 2022/Figure 2-figure supplement 1B-unlabeled and unedited image.tif]

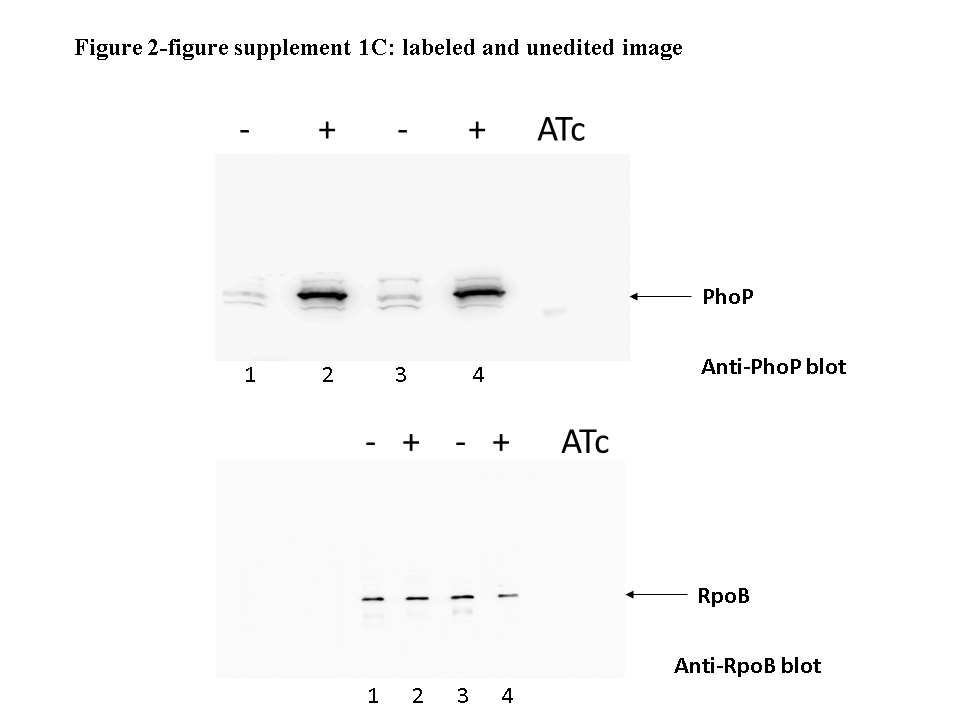

Supplement: Source data 1. [file elife-80965-data1.zip › Source Data File October 2022/Figure 2-figure supplement 1C-labeled and unedited image.tif]

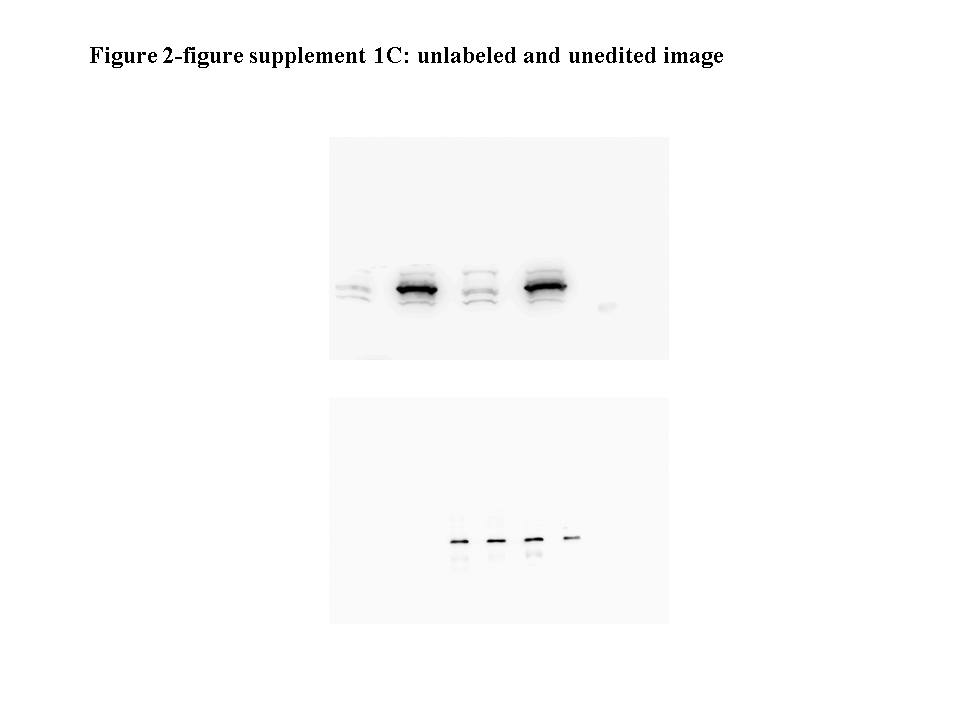

Supplement: Source data 1. [file elife-80965-data1.zip › Source Data File October 2022/Figure 2-figure supplement 1C-unlabeled and unedited image.tif]

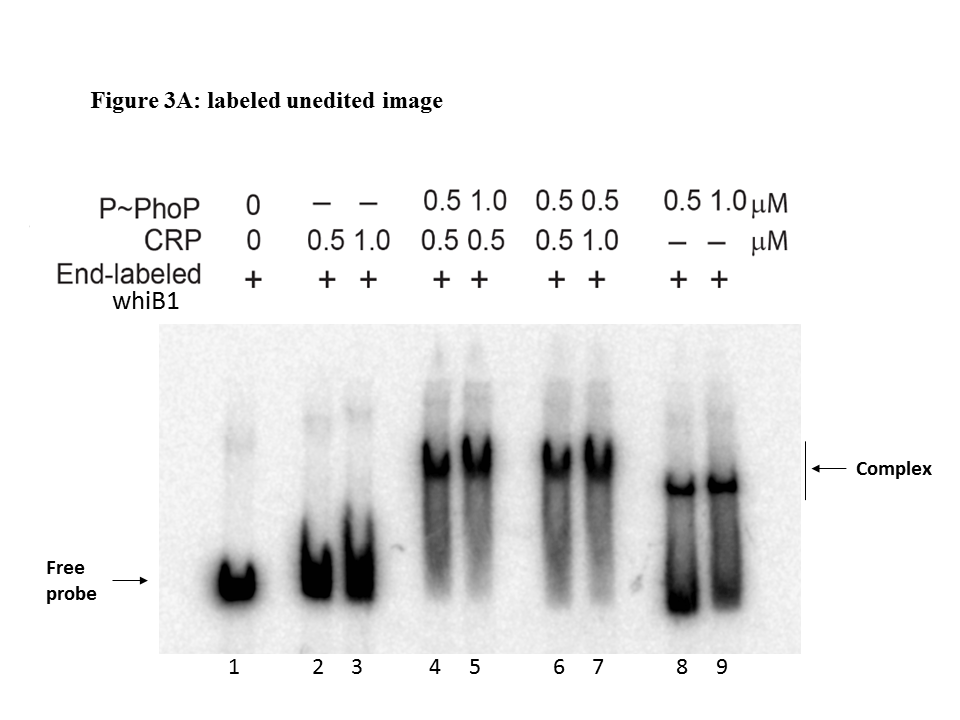

Supplement: Source data 1. [file elife-80965-data1.zip › Source Data File October 2022/Figure 3A-labeled unedited image.tif]

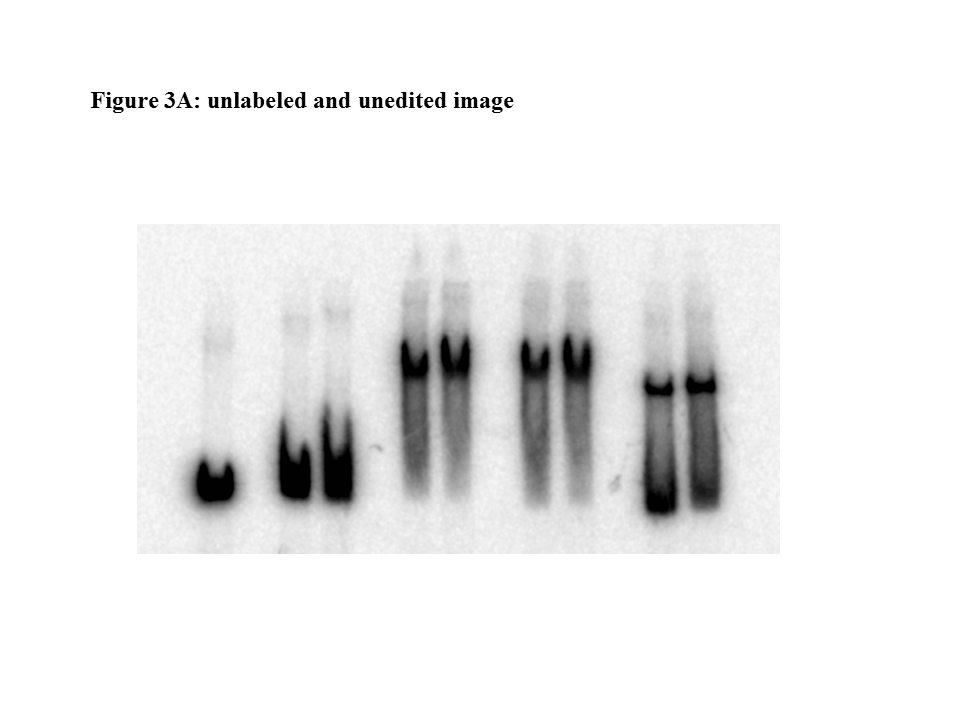

Supplement: Source data 1. [file elife-80965-data1.zip › Source Data File October 2022/Figure 3A-unlabeled and unedited image.tif]

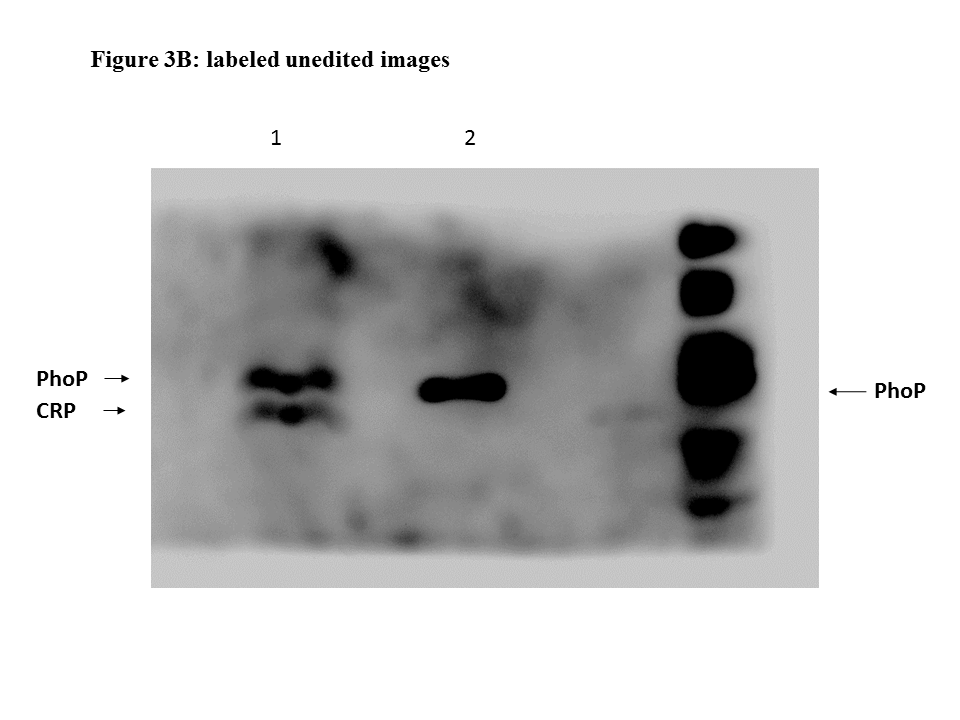

Supplement: Source data 1. [file elife-80965-data1.zip › Source Data File October 2022/Figure 3B - labeled unedited image.tif]

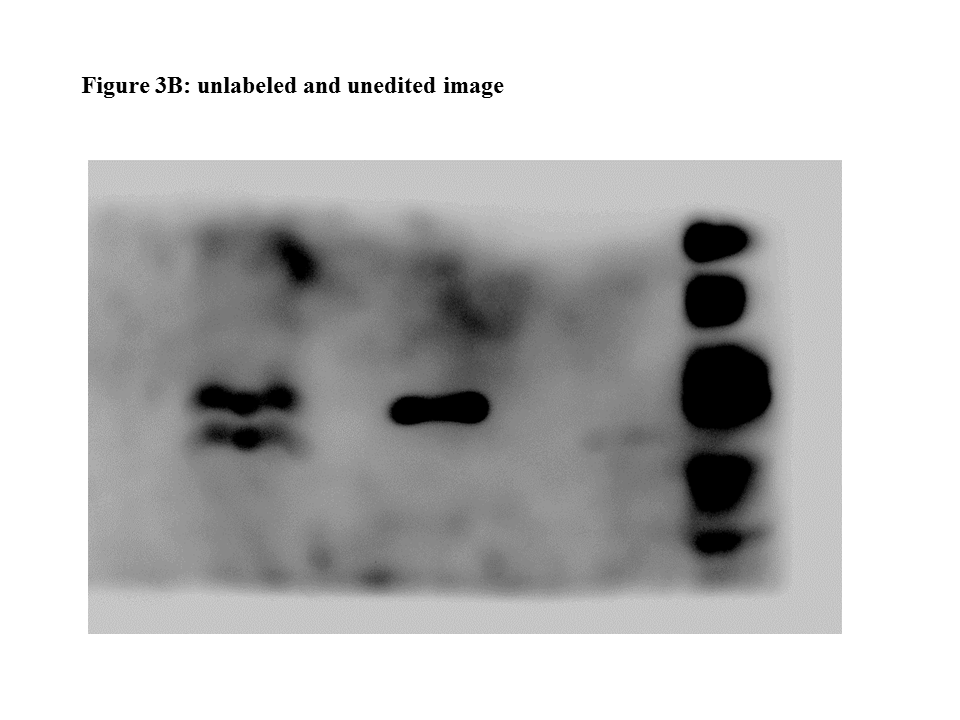

Supplement: Source data 1. [file elife-80965-data1.zip › Source Data File October 2022/Figure 3B-unlabeled and unedited image.tif]

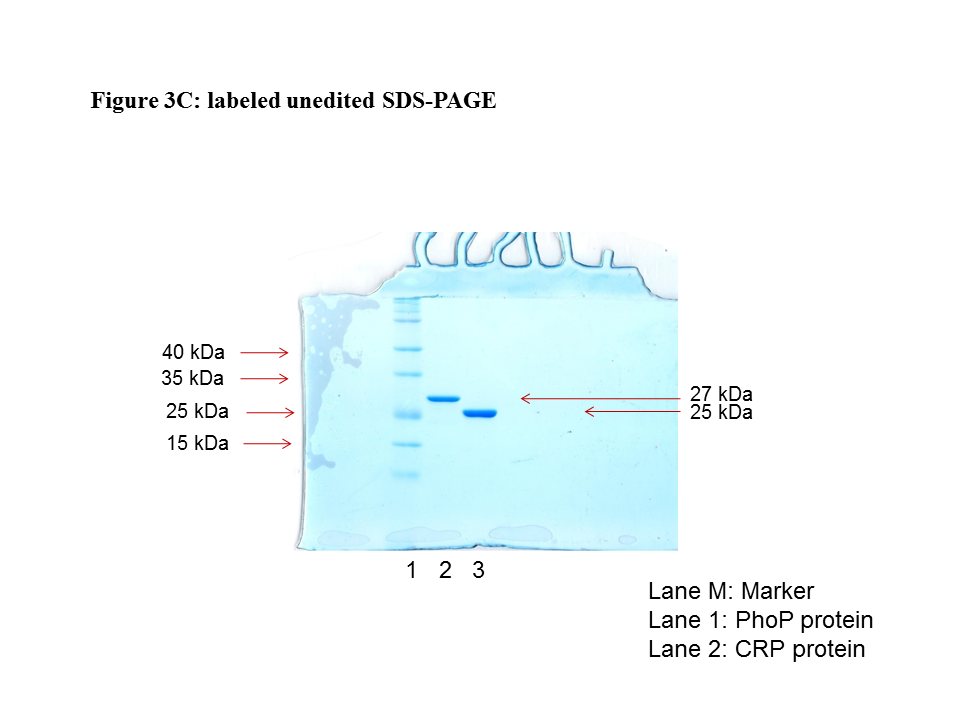

Supplement: Source data 1. [file elife-80965-data1.zip › Source Data File October 2022/Figure 3C-labeled unedited image.tif]

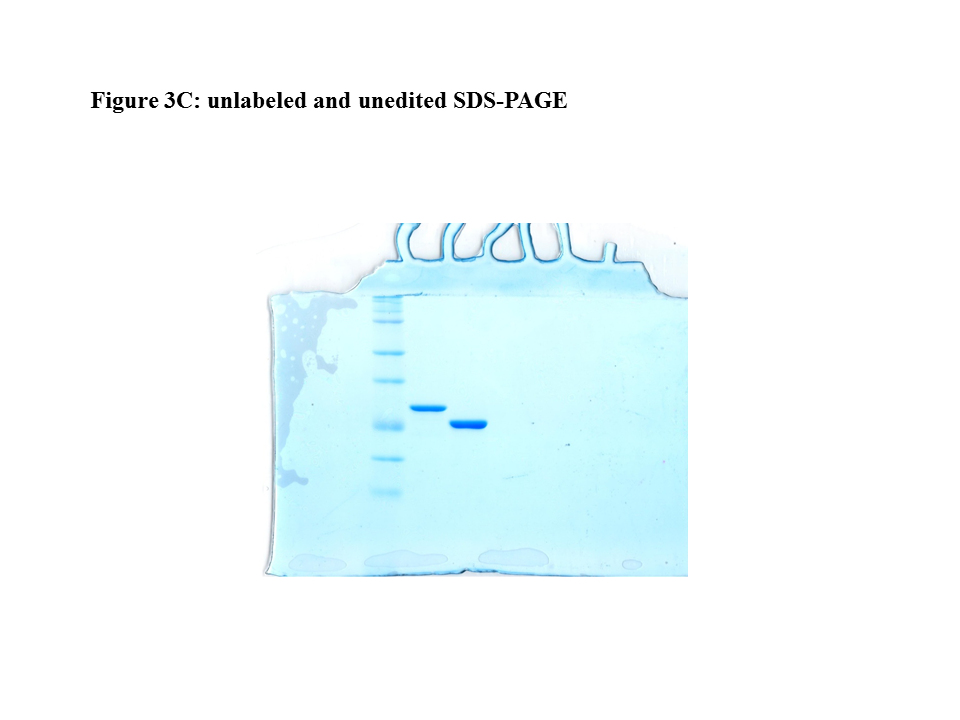

Supplement: Source data 1. [file elife-80965-data1.zip › Source Data File October 2022/Figure 3C-unlabeled and unedited image.tif]

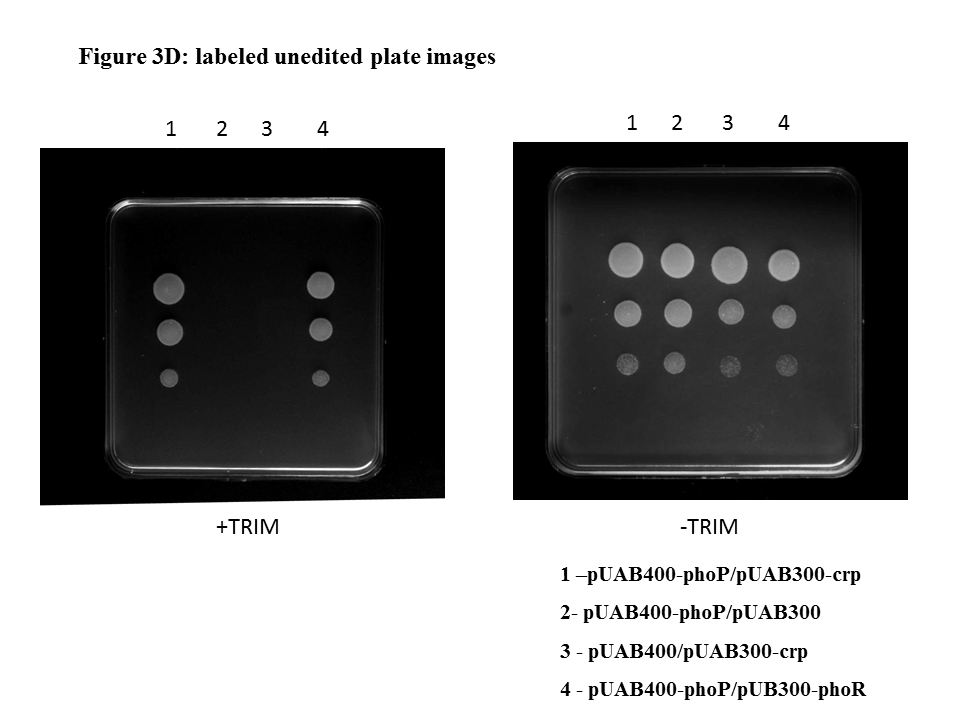

Supplement: Source data 1. [file elife-80965-data1.zip › Source Data File October 2022/Figure 3D-labeled unedited image.tif]

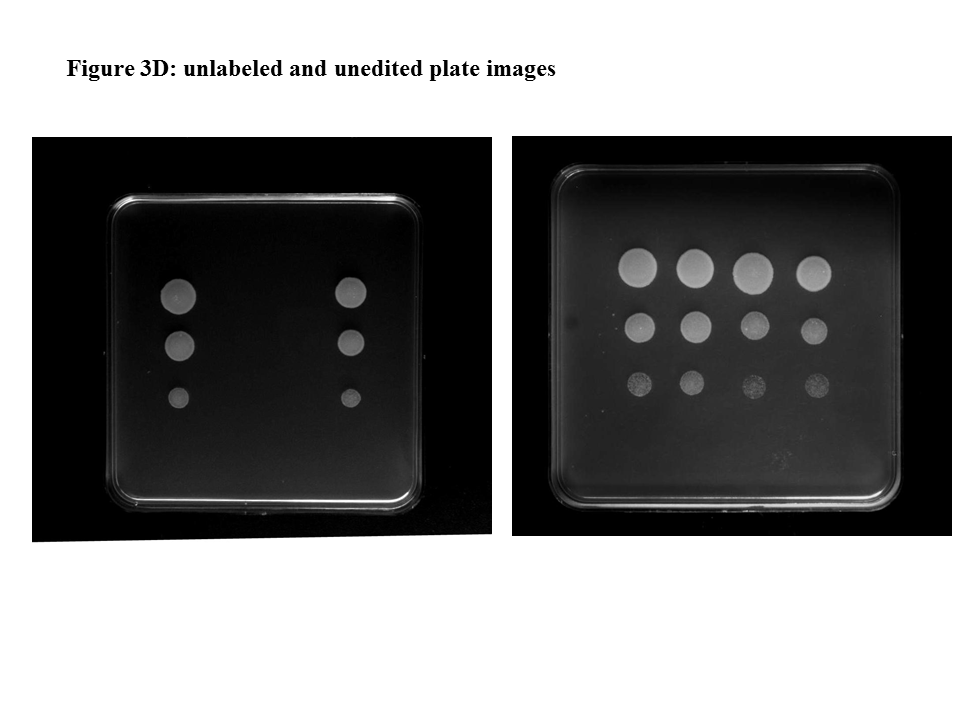

Supplement: Source data 1. [file elife-80965-data1.zip › Source Data File October 2022/Figure 3D-unlabeled and unedited image.tif]

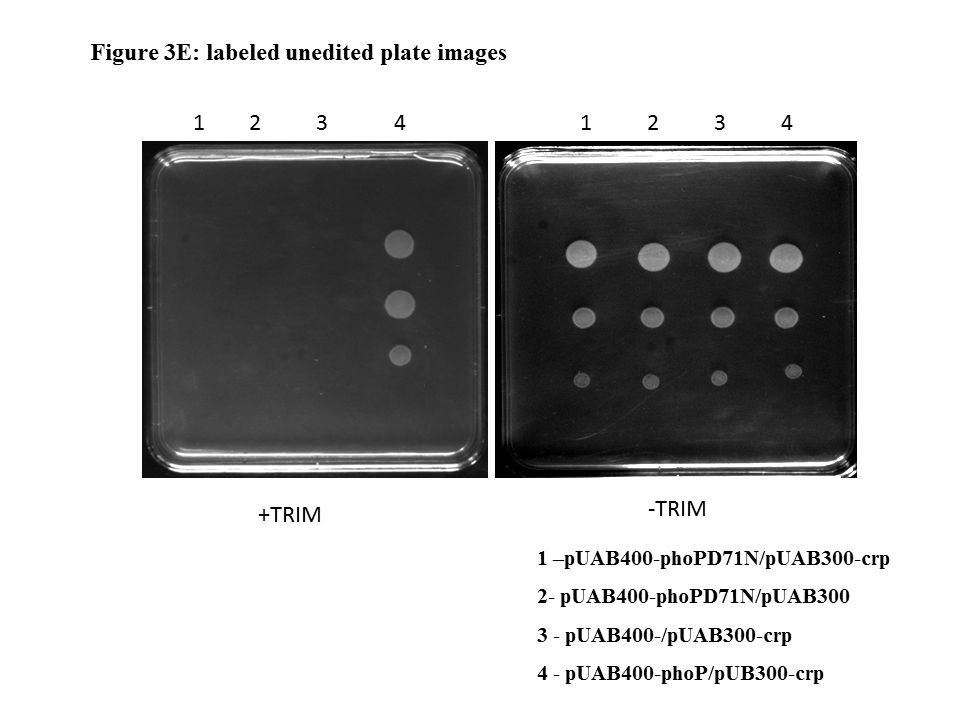

Supplement: Source data 1. [file elife-80965-data1.zip › Source Data File October 2022/Figure 3E-labeled unedited image.tif]

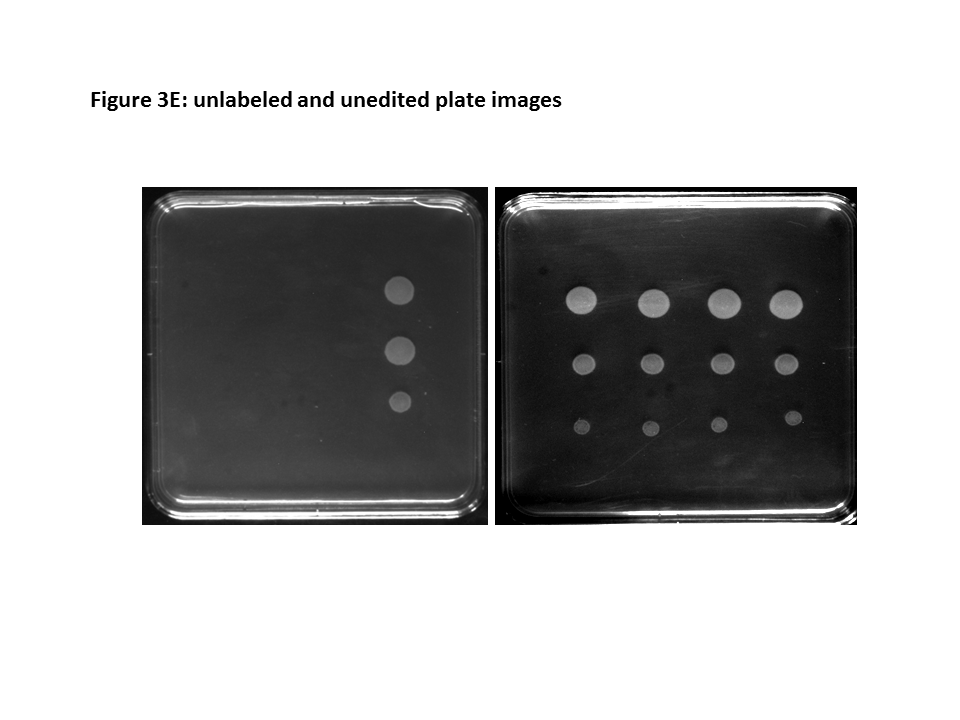

Supplement: Source data 1. [file elife-80965-data1.zip › Source Data File October 2022/Figure 3E-unlabeled and unedited image.tif]

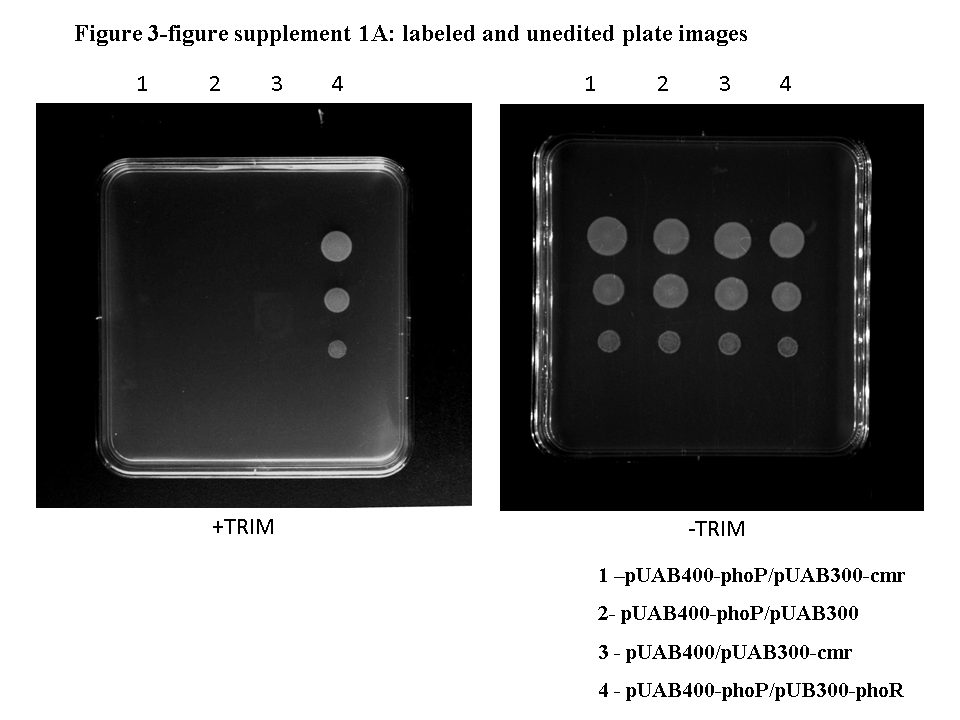

Supplement: Source data 1. [file elife-80965-data1.zip › Source Data File October 2022/Figure 3-figure supplement 1A-labeled and unedited plate images.tif]

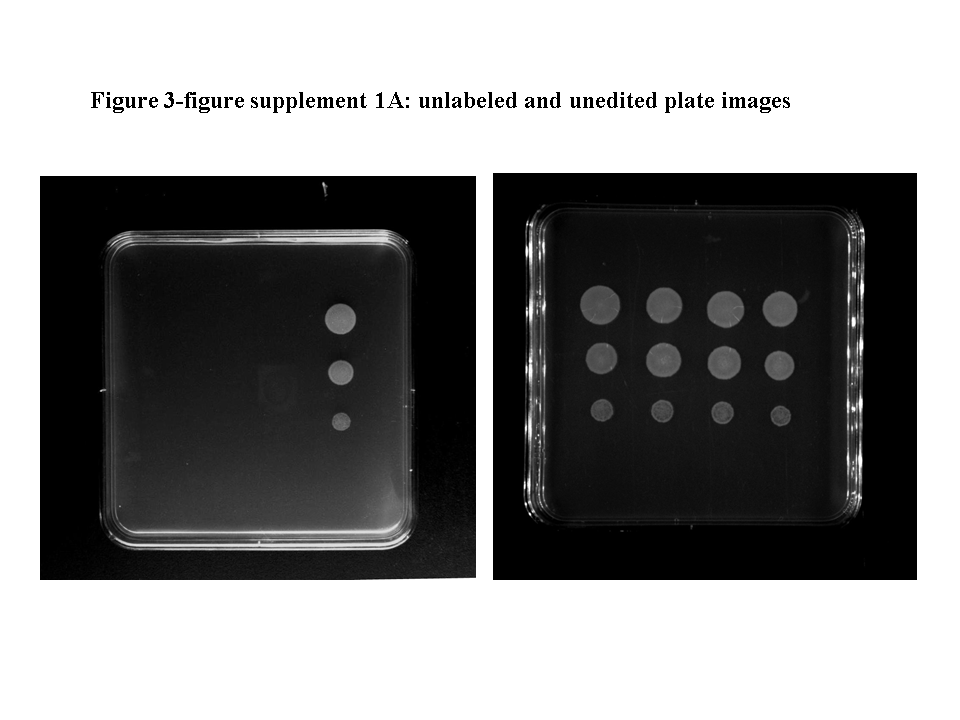

Supplement: Source data 1. [file elife-80965-data1.zip › Source Data File October 2022/Figure 3-figure supplement 1A-unlabeled and unedited plate images.tif]

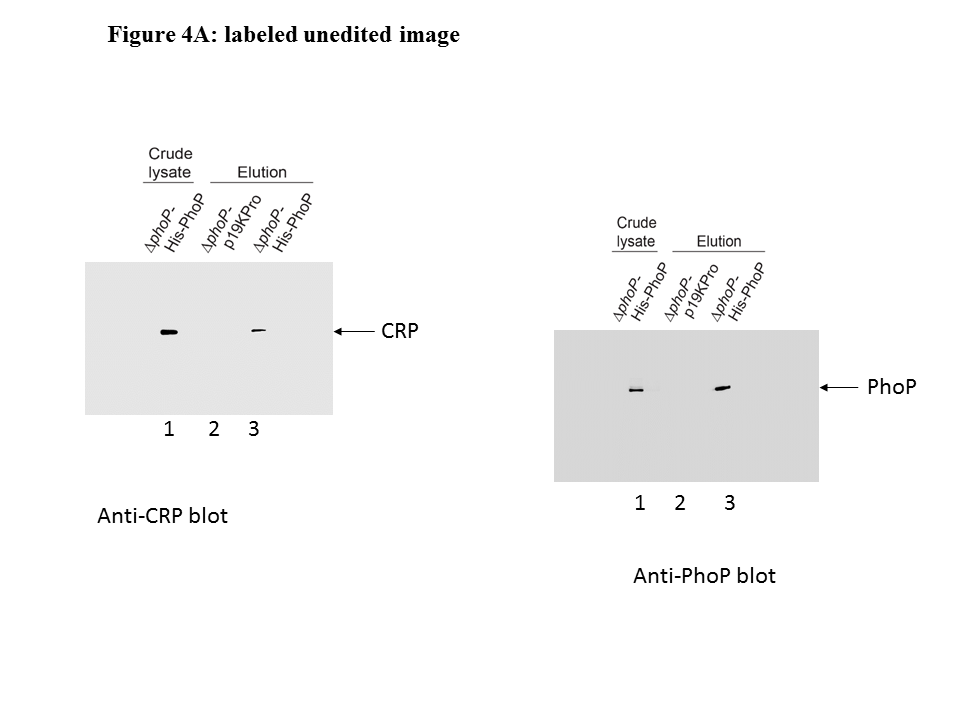

Supplement: Source data 1. [file elife-80965-data1.zip › Source Data File October 2022/Figure 4A- labeled unedited image.tif]

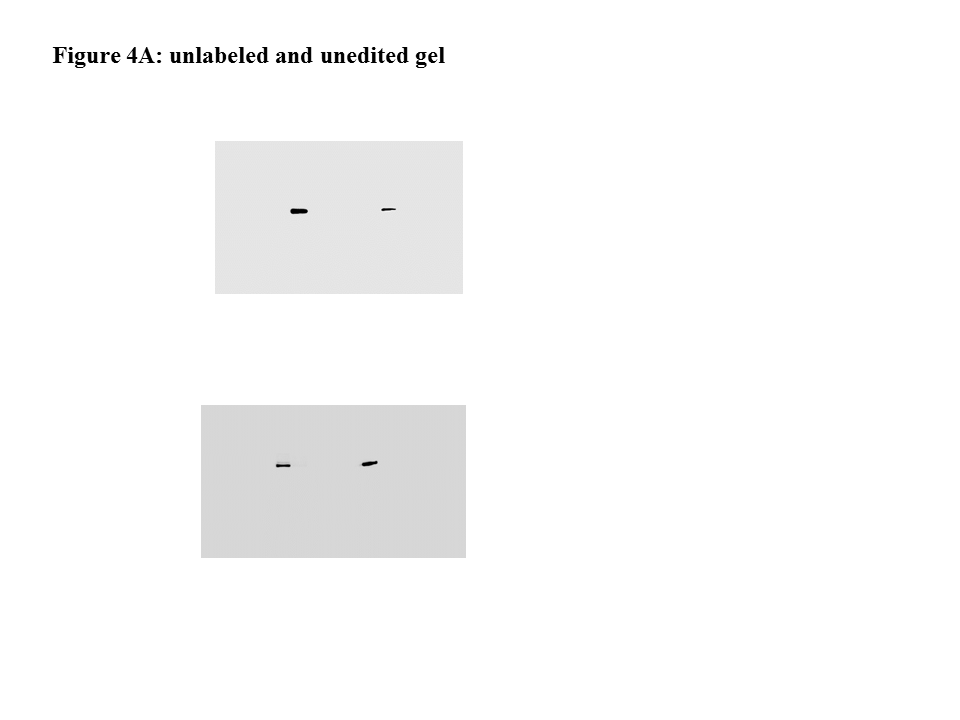

Supplement: Source data 1. [file elife-80965-data1.zip › Source Data File October 2022/Figure 4A-unlabeled and unedited image.tif]

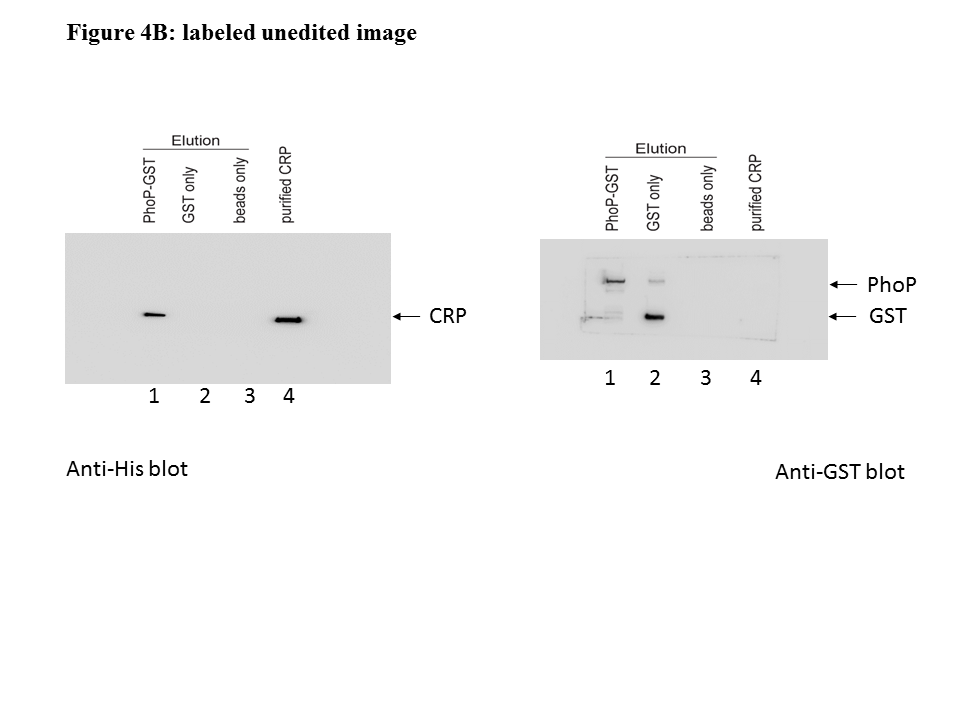

Supplement: Source data 1. [file elife-80965-data1.zip › Source Data File October 2022/Figure 4B -labeled unedited image.tif]

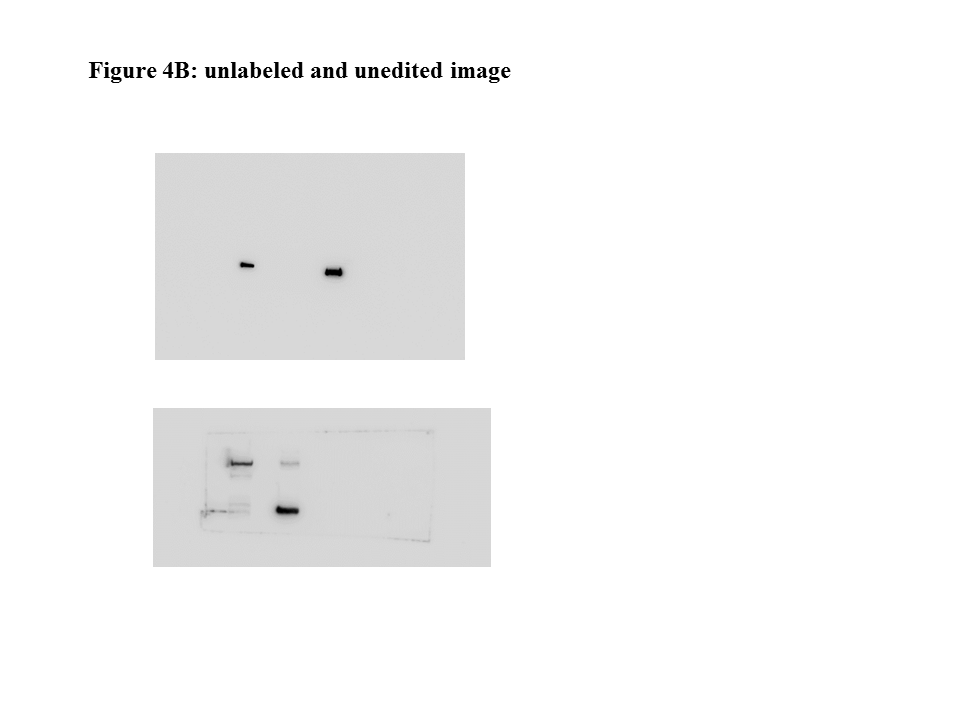

Supplement: Source data 1. [file elife-80965-data1.zip › Source Data File October 2022/Figure 4B-unlabeled and unedited image.tif]

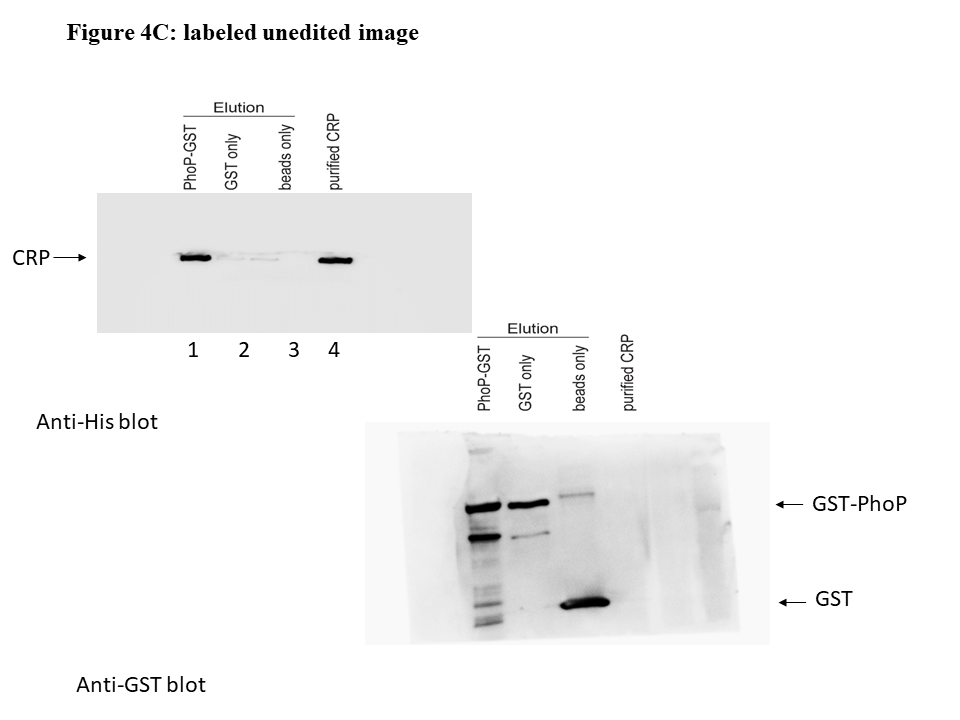

Supplement: Source data 1. [file elife-80965-data1.zip › Source Data File October 2022/Figure 4C- labeled unedited image.tif]

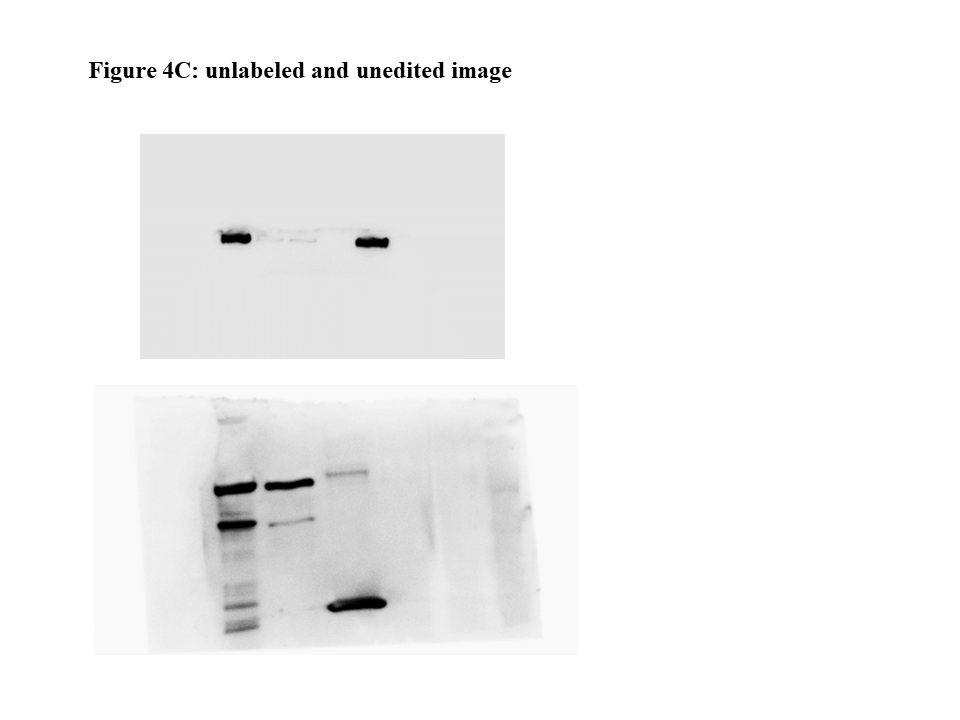

Supplement: Source data 1. [file elife-80965-data1.zip › Source Data File October 2022/Figure 4C-unlabeled and unedited image.tif]

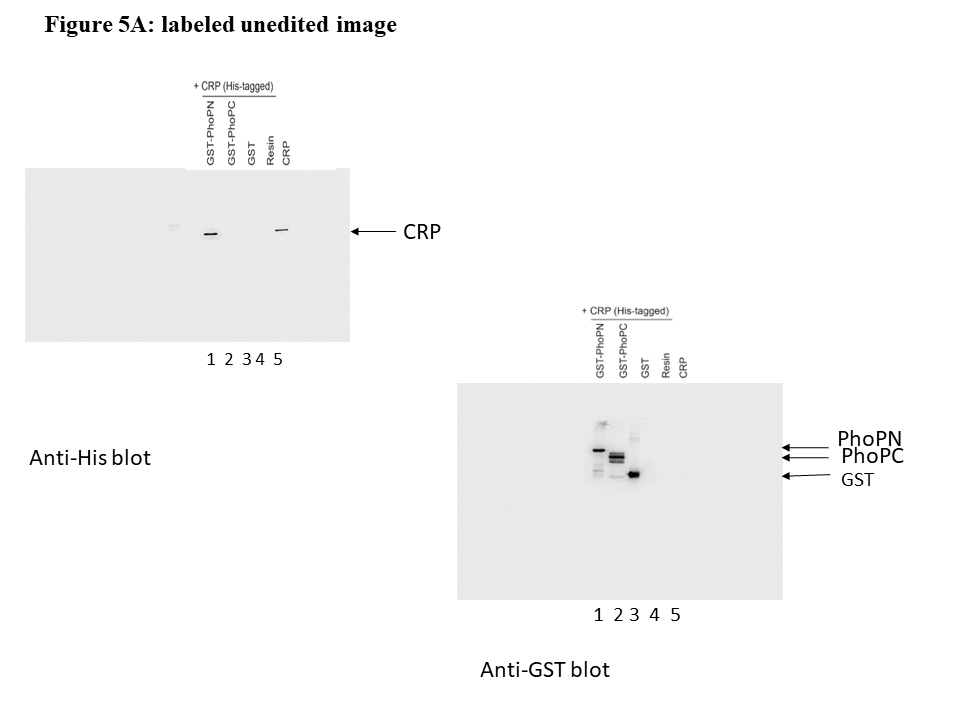

Supplement: Source data 1. [file elife-80965-data1.zip › Source Data File October 2022/Figure 5A -labeled unedited image.tif]

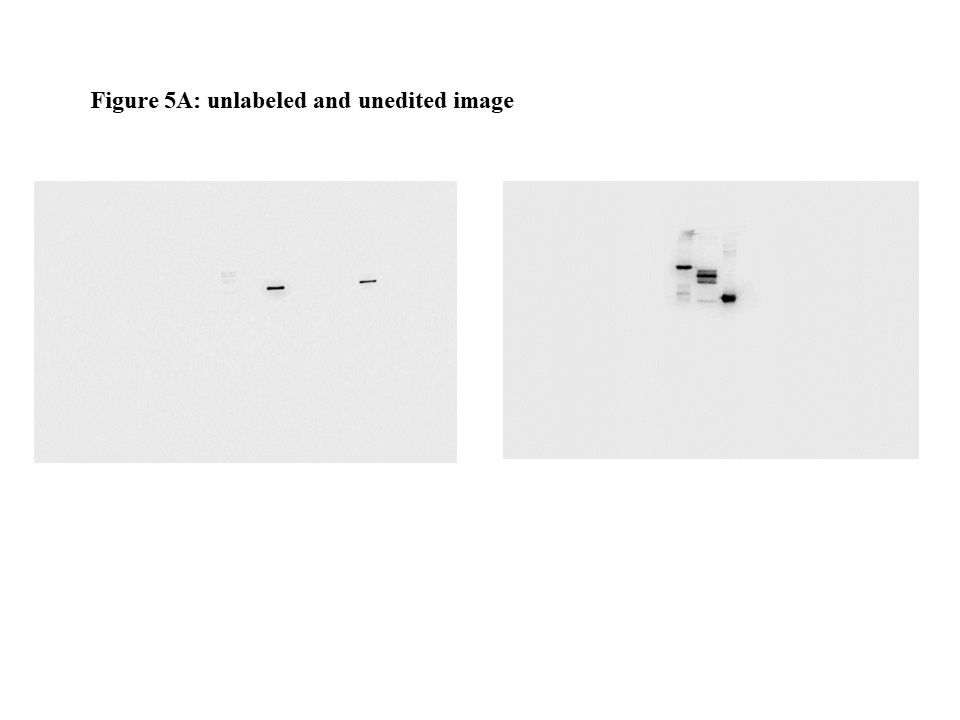

Supplement: Source data 1. [file elife-80965-data1.zip › Source Data File October 2022/Figure 5A-unlabeled and unedited image.tif]

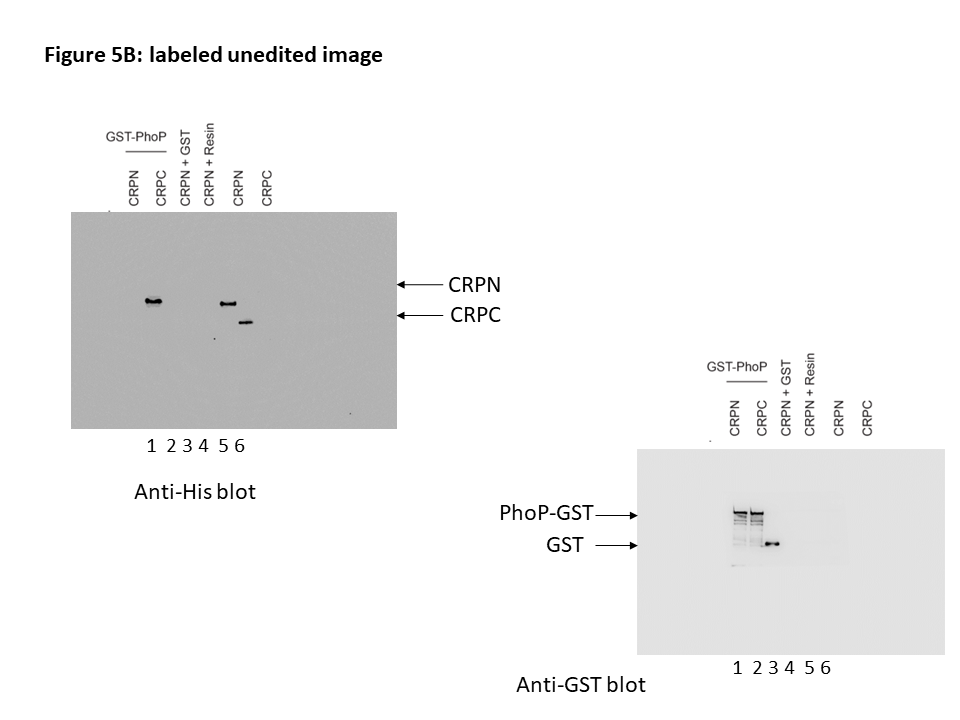

Supplement: Source data 1. [file elife-80965-data1.zip › Source Data File October 2022/Figure 5B-labeled unedited image.tif]

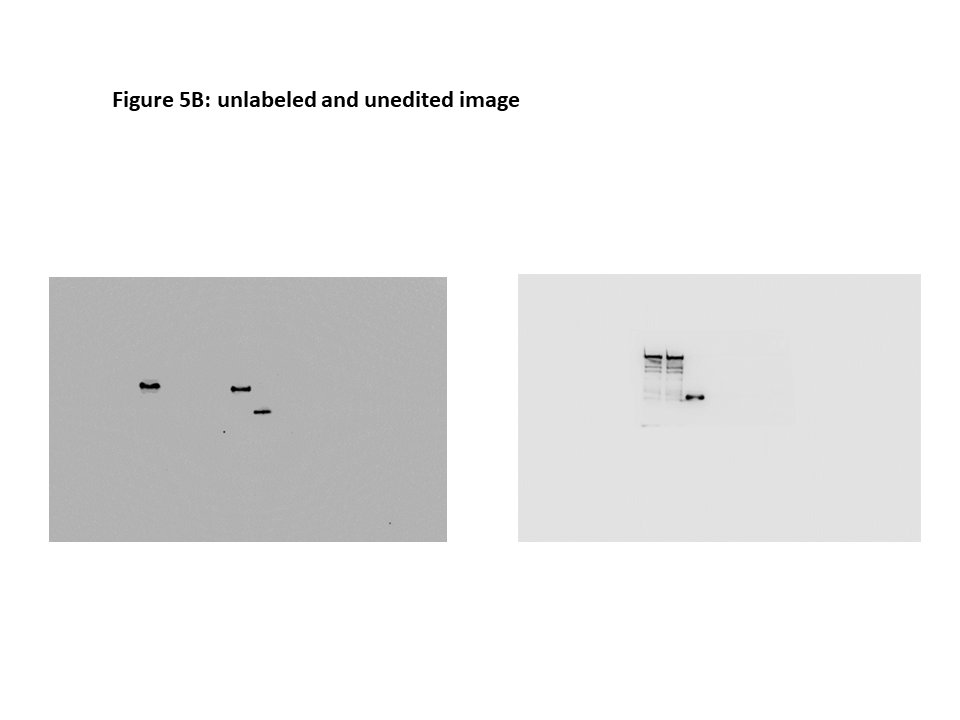

Supplement: Source data 1. [file elife-80965-data1.zip › Source Data File October 2022/Figure 5B-unlabeled and unedited image.tif]

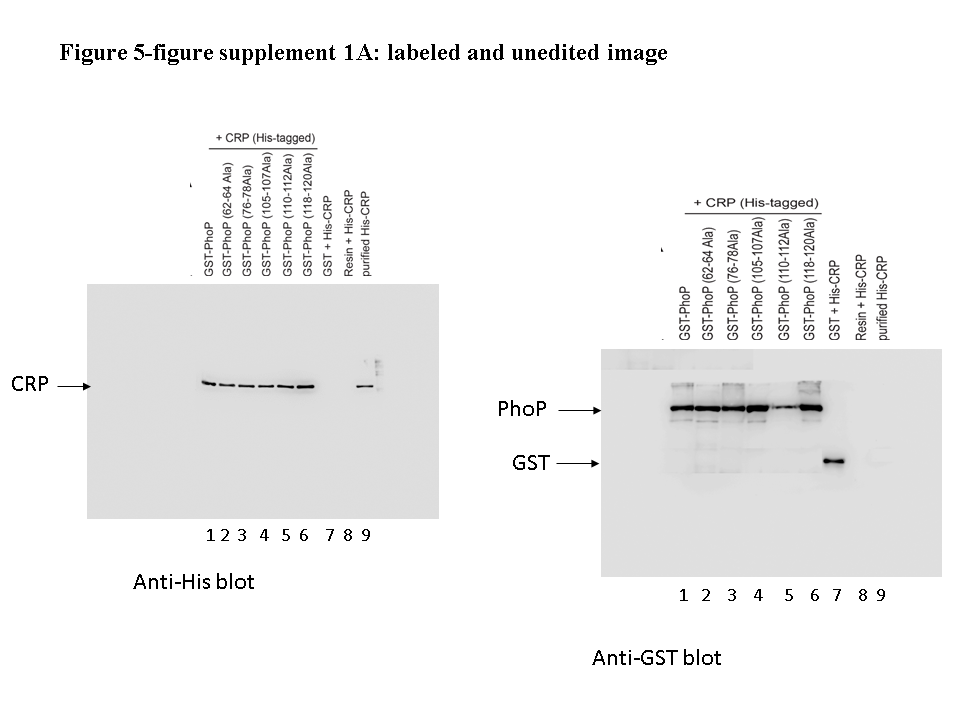

Supplement: Source data 1. [file elife-80965-data1.zip › Source Data File October 2022/Figure 5-figure supplement 1A-labeled and unedited image.tif]

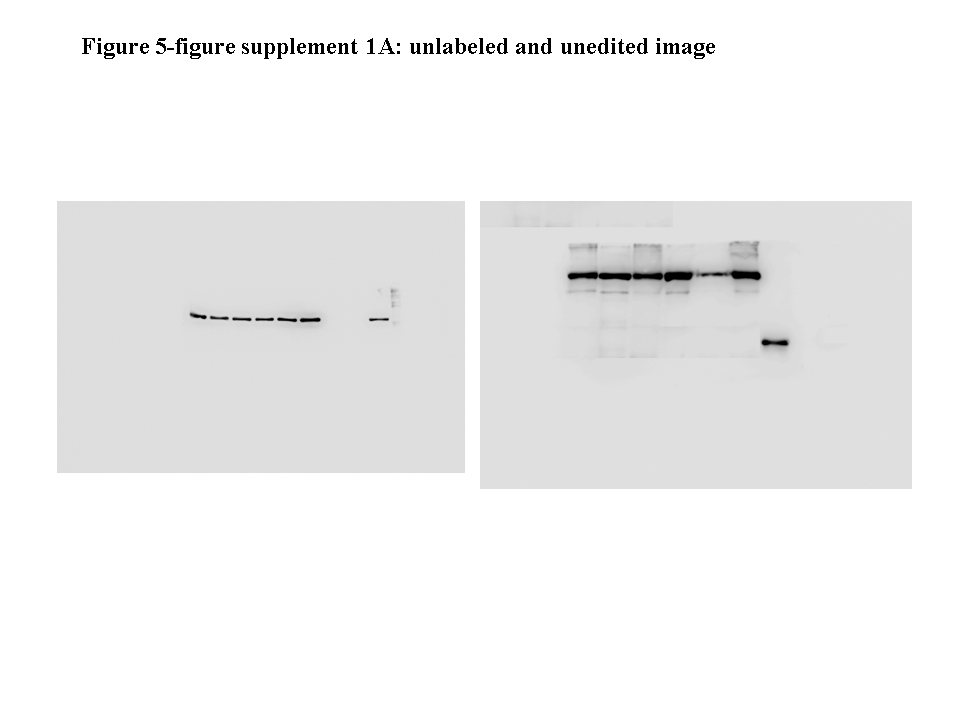

Supplement: Source data 1. [file elife-80965-data1.zip › Source Data File October 2022/Figure 5-figure supplement 1A-unlabeled and unedited image.tif]

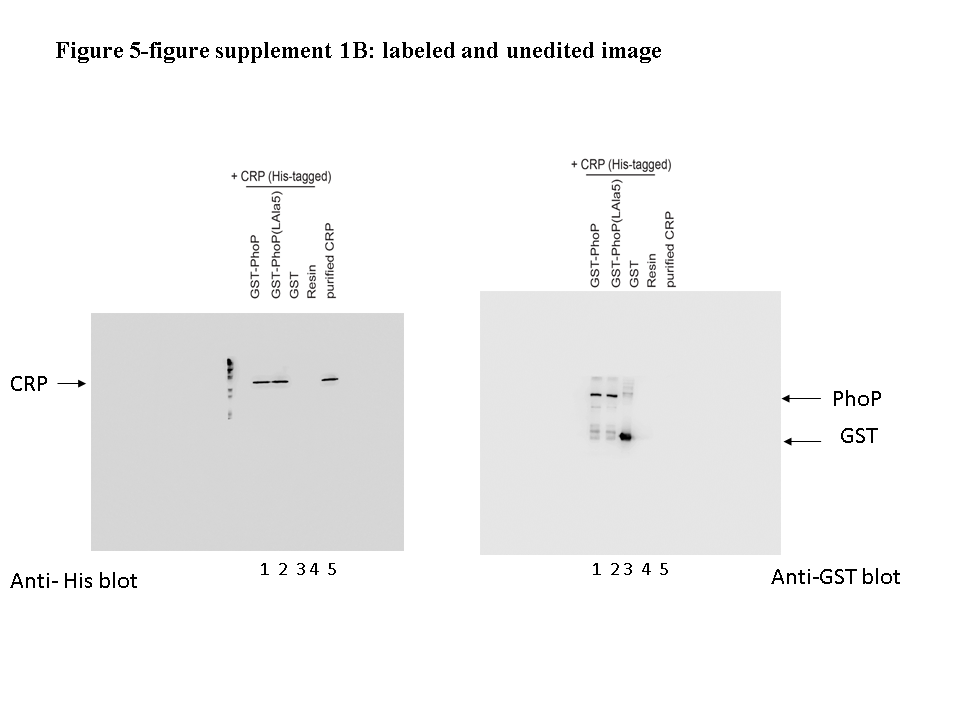

Supplement: Source data 1. [file elife-80965-data1.zip › Source Data File October 2022/Figure 5-figure supplement 1B-labeled and unedited image.tif]

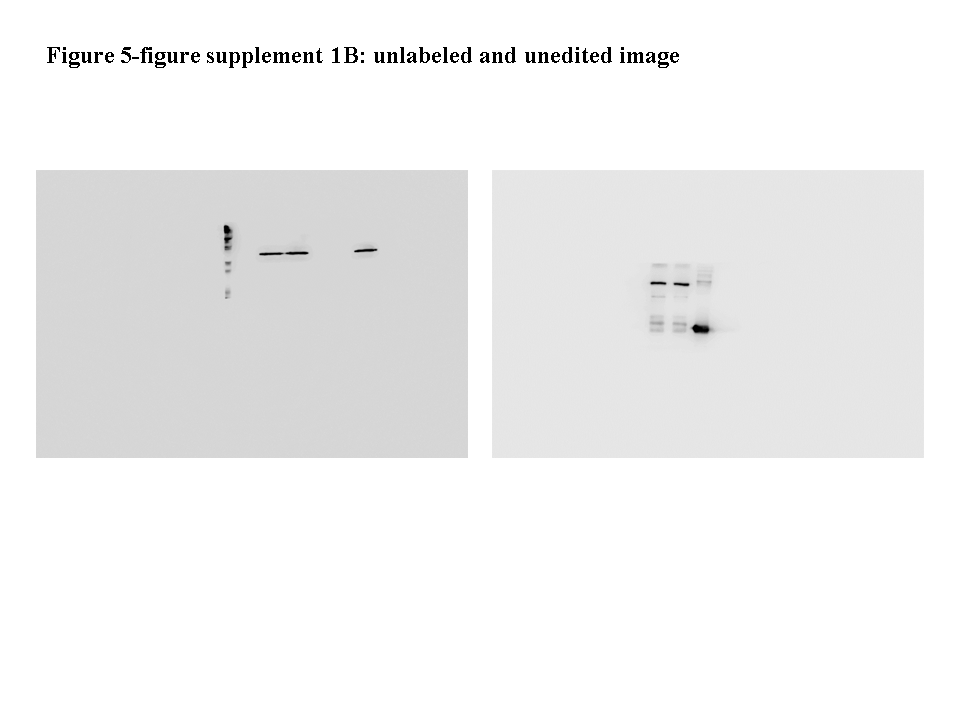

Supplement: Source data 1. [file elife-80965-data1.zip › Source Data File October 2022/Figure 5-figure supplement 1B-unlabeled and unedited image.tif]

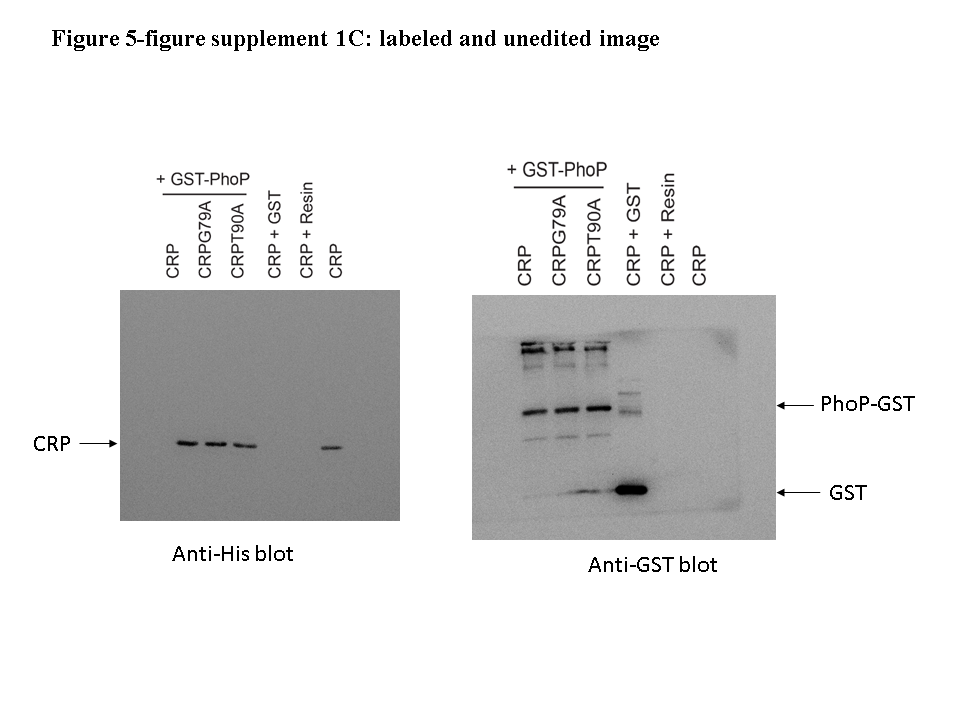

Supplement: Source data 1. [file elife-80965-data1.zip › Source Data File October 2022/Figure 5-figure supplement 1C-labeled and unedited image.tif]

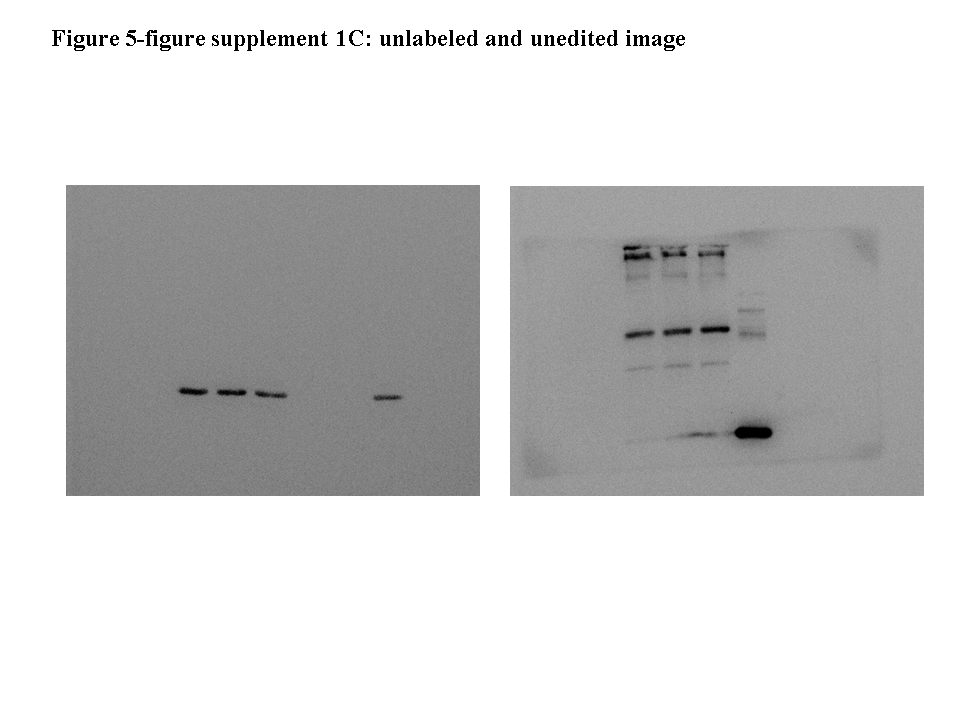

Supplement: Source data 1. [file elife-80965-data1.zip › Source Data File October 2022/Figure 5-figure supplement 1C-unlabeled and unedited image.tif]

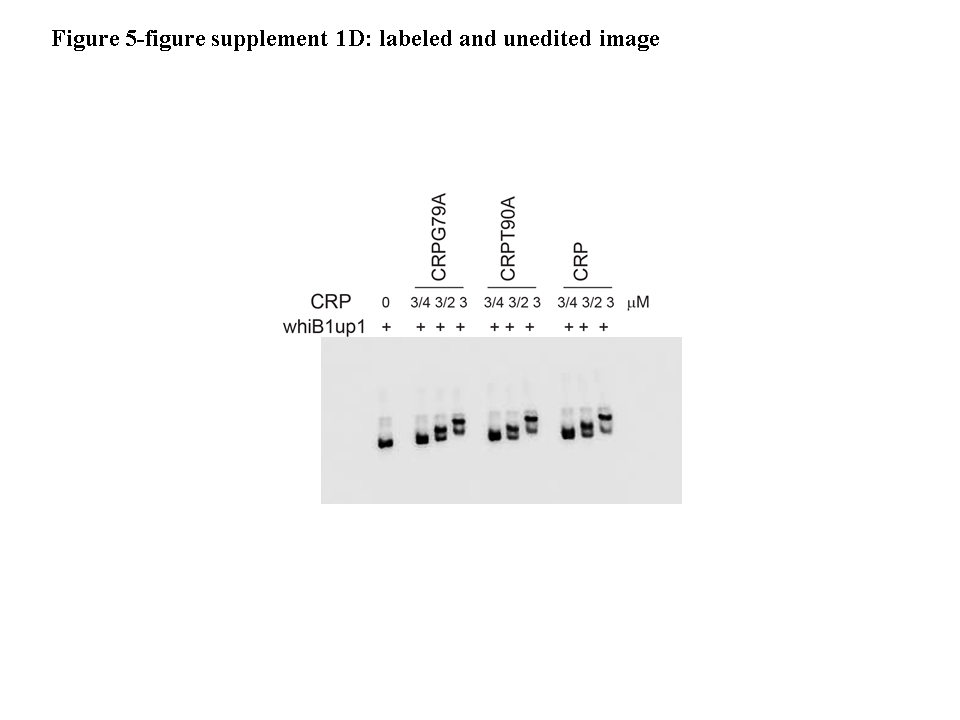

Supplement: Source data 1. [file elife-80965-data1.zip › Source Data File October 2022/Figure 5-figure supplement 1D-labeled and unedited image.tif]

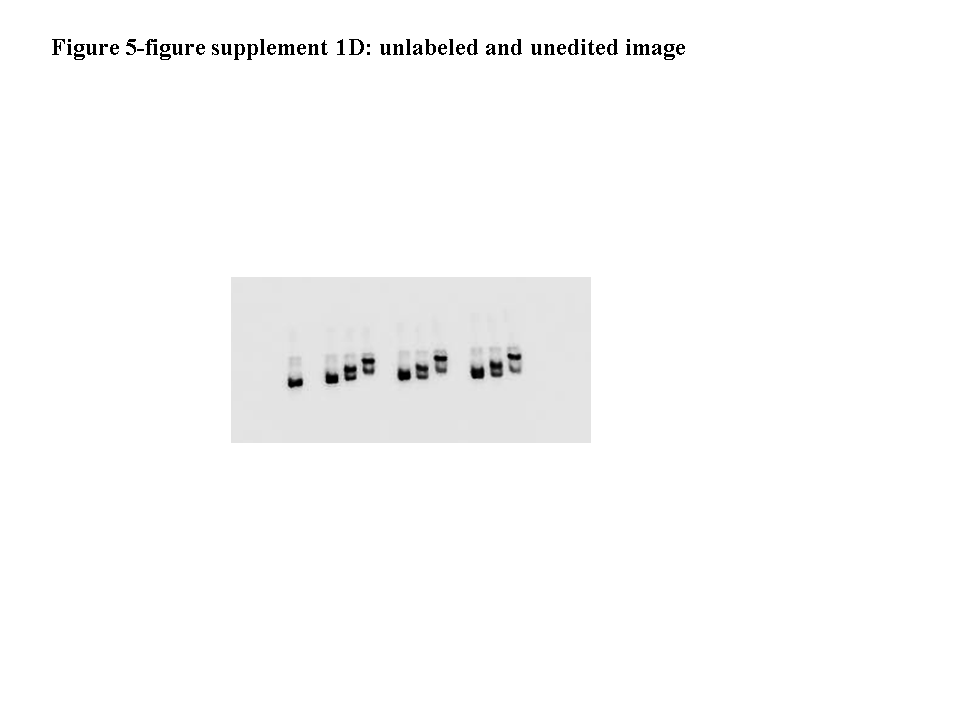

Supplement: Source data 1. [file elife-80965-data1.zip › Source Data File October 2022/Figure 5-figure supplement 1D-unlabeled and unedited image.tif]

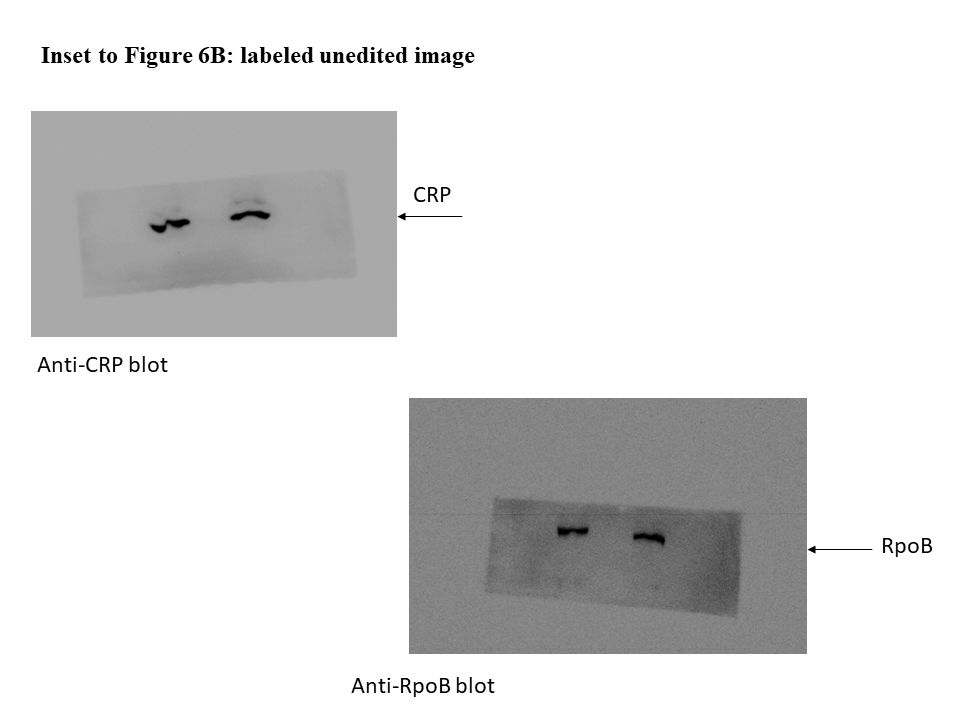

Supplement: Source data 1. [file elife-80965-data1.zip › Source Data File October 2022/Inset to Figure 6B inset-labeled and unedited image.tif]

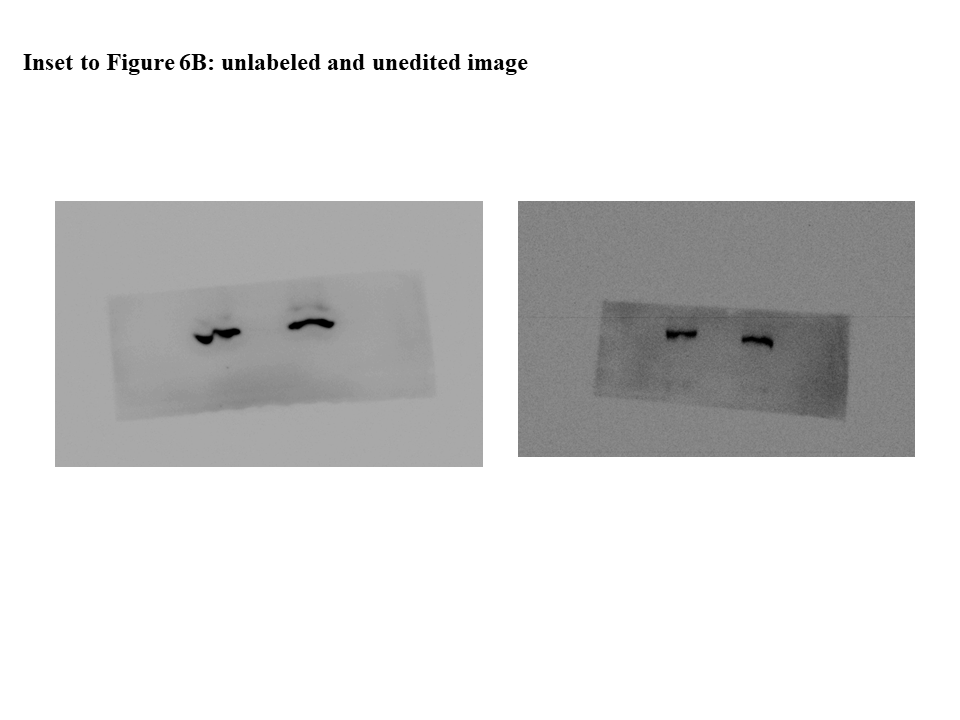

Supplement: Source data 1. [file elife-80965-data1.zip › Source Data File October 2022/Inset to Figure 6B-unlabeled and unedited image.tif]
